# Recent Development in Intelligent Compaction for Asphalt Pavement Construction: Leveraging Smart Sensors and Machine Learning

**DOI:** 10.3390/s24092777

**Published:** 2024-04-26

**Authors:** Yudan Wang, Jue Li, Xinqiang Zhang, Yongsheng Yao, Yi Peng

**Affiliations:** 1 School of Civil Engineering, Chongqing Jiaotong University, Chongqing 400074, China; 2 College of Traffic & Transportation, Chongqing Jiaotong University, Chongqing 400074, China

**Keywords:** pavement engineering, intelligent compaction, quality evaluation, smart sensor, machine learning

## Abstract

Intelligent compaction (IC) has emerged as a breakthrough technology that utilizes advanced sensing, data transmission, and control systems to optimize asphalt pavement compaction quality and efficiency. However, accurate assessment of compaction status remains challenging under real construction conditions. This paper reviewed recent progress and applications of smart sensors and machine learning (ML) to address existing limitations in IC. The principles and components of various advanced sensors deployed in IC systems were introduced, including SmartRock, fiber Bragg grating, and integrated circuit piezoelectric acceleration sensors. Case studies on utilizing these sensors for particle behavior monitoring, strain measurement, and impact data collection were reviewed. Meanwhile, common ML algorithms including regression, classification, clustering, and artificial neural networks were discussed. Practical examples of applying ML to estimate mechanical properties, evaluate overall compaction quality, and predict soil firmness through supervised and unsupervised models were examined. Results indicated smart sensors have enhanced compaction monitoring capabilities but require robustness improvements. ML provides a data-driven approach to complement traditional empirical methods but necessitates extensive field validation. Potential integration with digital construction technologies such as building information modeling and augmented reality was also explored. In conclusion, leveraging emerging sensing and artificial intelligence presents opportunities to optimize the IC process and address key challenges. However, cooperation across disciplines will be vital to test and refine technologies under real-world conditions. This study serves to advance understanding and highlight priority areas for future research toward the realization of IC’s full potential.

## 1. Introduction

In recent years, the rapid progress of highway construction in China has been propelled by economic and societal advancements. The utilization of asphalt pavement has been extensive in these highway construction projects due to its durability, cost-effectiveness, and adaptability to various climates [1,2,3]. However, subjected to heavy traffic loads and environmental factors, inadequately compacted asphalt pavement may develop defects such as cracks, ruts, and pits over time [4]. These issues significantly affect both traffic safety and driving comfort, making it imperative to address them to enhance the pavement performance. Among the numerous factors contributing to the substandard quality of asphalt pavement, non-compliance with compaction standards plays a crucial role [5]. As the final phase of the construction process, compaction assumes paramount importance in ensuring the compactness and performance of asphalt mixture [6]. Consequently, IC technology has emerged as a viable solution that can improve the durability and compaction quality of asphalt pavements. Moreover, it enables accurate quality monitoring and timely detection of defects, thus resulting in saved construction time. Although current research in asphalt pavement has predominantly focused on exploring new materials, structures, and design methods, the significance of IC research in paving technology should not be overlooked.

Conventional compaction quality inspection methods for asphalt pavement, such as sand filling and water filling, measure the mass and volume of the samples taken on site, calculate the density, and then compare it with the standard maximum dry density to obtain the compaction degree. However, there are limitations in terms of speed and accuracy. The sand filling method is renowned for its slow operation, while the water filling method lacks accuracy in determining compaction quality [7]. Moreover, compaction quality management practices for asphalt pavement often rely on a post-event sampling inspection approach, which presents challenges in promptly assessing the compaction status and ensuring overall quality control [8]. Although traditional compaction equipment and techniques have been instrumental in attaining the desired level of compaction in road materials over the years, they encounter significant challenges. Firstly, risks for inspectors are introduced and potential disruptions to subsequent construction operations may arise due to the reliance on field measurements in traditional methods [9,10,11]. Secondly, the limited number of test samples fails to provide a comprehensive representation of compaction quality across the entire area [12,13,14], thereby hindering accurate evaluation and uniformity assurance [15]. In addition, the correlation between the pavement density and the device reading is essential to be established before compaction, making it less convenient and less applicable to different materials, especially the materials with different design and materials sources. Lastly, the absence of real-time feedback capabilities in traditional pressure equipment makes it difficult for operators to make timely adjustments, potentially resulting in issues such as over-compaction or inadequate compaction [9,16]. The inherent flaws in conventional compaction practices have various implications, including diminished efficiency, increased costs, compromised pavement performance, shortened road service life, and augmented expenses for maintenance and repair in road construction projects. Conversely, IC technology emerges as a promising alternative. By incorporating advanced sensing, control, and monitoring systems, IC offers several advantages over traditional compaction methods. Real-time feedback enables operators to make precise adjustments, ensuring optimal compaction while minimizing risks of over-compaction or inadequate compaction. Additionally, the ability to collect comprehensive data on compaction uniformity enhances quality control and leads to improved pavement performance and an extended service life. Therefore, the adoption of IC technology represents a significant advancement in the field of compaction quality management for asphalt pavement. By addressing the limitations of traditional methods, improved efficiency and enhanced quality control are made possible through IC.

In the era of Industry 4.0, IC technology has undergone a revolutionary transformation with the introduction of advanced technologies including smart sensors and ML [17,18,19,20,21]. The integration of modern information technology, particularly artificial intelligence, with the traditional construction industry has become an inevitable outcome [14,22,23]. The benefits brought by Industry 4.0 and digitalization are harnessed, leading to the transformation of the construction industry through IC technology. As defined by the Federal Highway Administration (FHWA), IC technology is an advanced compaction that utilizes rollers equipped with state-of-the-art measurement systems, including accelerometers, global positioning systems (GPS), infrared thermometers, and on-board reporting systems [24]. By deploying these technologies, a comprehensive and data-driven compaction control approach is achieved, shifting compaction quality management from post-inspection to a proactive methodology [25,26,27]. Through the integration of compaction monitoring systems and remote monitoring technologies, IC ensures the timely detection and mitigation of issues including under-compaction and over-compaction, resulting in improved pavement performance [28]. Additionally, the utilization of precise compaction cycles within the IC system can enhance the quality of road construction and minimize energy consumption, thereby reflecting the positive influence of industrialization, informationization, and intelligence on modern expressway development [29,30,31]. The convergence of IC technology with Industry 4.0 principles and digitalization trends enables the comprehensive recording, sharing, and analysis of valuable data related to paving materials, construction equipment, and techniques [32]. This data-driven approach empowers pavement operations, management, maintenance, and repair by providing a solid foundation for informed decision making and the implementation of proactive maintenance strategies [33]. Furthermore, IC technology has ushered in a new era of real-time temperature detection and evaluation of pavement quality and uniformity [30]. This capability has significantly contributed to the widespread acceptance and adoption of IC throughout the pavement industry.

Consequently, IC holds great promise for development in pavement engineering. This study conducted a comprehensive review to facilitate a deep understanding of IC technology. The principles, components, development, and application of IC technology were introduced. It emphasized the significance of IC as a real-time and comprehensive quality monitoring method that can effectively address the increasing demands and challenges faced in the pavement construction industry. The findings of this study have the potential to guide decision-making processes regarding the implementation and utilization of IC technology. The knowledge gained from this research can drive further advancements in pavement compaction, ultimately leading to enhanced construction processes, improved quality management, and increased efficiency in the industry.

## 2. Technical Principle and Technical Characteristics of Intelligent Compaction

IC is a sophisticated technology that achieves automatic control and precise monitoring of compaction machinery through integration of advanced sensing devices, communication networks, and digital control systems. Compared to traditional practices reliant on manual operation and periodic sampling, IC represents a paradigm shift toward continuous, real-time management of the entire compaction process from start to finish. This section provides an in-depth examination of IC’s underlying mechanisms, core components, and distinguishing attributes that differentiate it from conventional techniques.

### 2.1. Proposition of Intelligent Compaction

Prior to 2000, the progress and widespread adoption of IC technology were hindered by both societal and technical limitations, resulting in slow advancement [34]. However, in 2002, the FHWA and American Association of State Highway and Transportation Officials (AASHTO) took proactive measures by dispatching a team to Europe to investigate the development of IC technology [28]. This marked a significant turning point, leading to the increased attention and practical application of IC research in the United States [35]. The National Intelligent Compaction Project was jointly launched by the FHWA and the transportation departments of 12 states in the United States in 2007 [36]. This project focused on a comprehensive study of IC technology for asphalt pavement. Each participating state conducted demonstration projects, evaluated and analyzed IC technology, and formulated IC standards and guidelines. The resulting systematic IC standards are presented in Table 1. Since then, research on IC has steadily grown and garnered increasing attention, particularly from 2010 onwards [37]. Notably, construction equipment manufacturers such as BOMAG in Boppard, Germany, Dynapac in Stockholm, Sweden, Caterpillar in IL, USA, and Sakai in Japan have made significant contributions to IC research [15,27].

Although previous investigations into IC predominantly focused on soil compaction and control of soil–rock mixtures, there has been relatively less emphasis on its application in compacting asphalt pavements. While the study of soil compaction serves as a valuable reference for research on asphalt pavement compaction, it is crucial to acknowledge the substantial disparities between the two regarding their structural composition and performance requirements, necessitating dedicated attention.

### 2.2. Technical Principle and Composition of Intelligent Compaction

**Figure 1:**
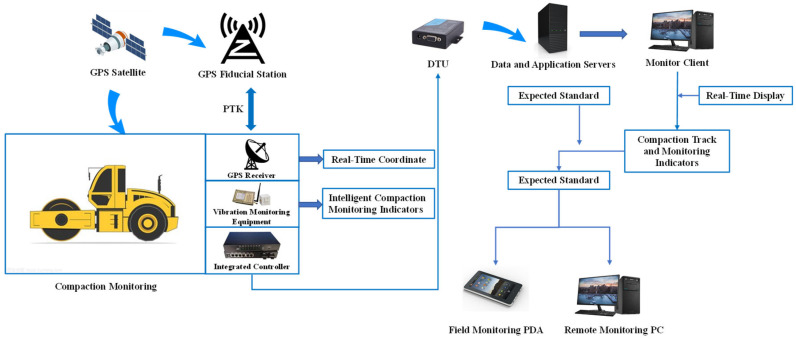
IC and continuous detection technology based on GPS System.

IC technology employs vibratory rollers equipped with advanced features such as real-time dynamic GPS, roller integrated measurement systems, feedback control mechanisms, and on-board real-time display of all IC measurements. These rollers are utilized for compacting various materials including soil, aggregates, and asphalt mixtures [38,39,40]. The integration of precision sensing technology, computer technology, and high-precision positioning technology has given rise to a groundbreaking compaction technology, as illustrated in Figure 1. This technology enables seamless control of compaction equipment and facilitates instantaneous transmission of real-time information to operators [41].

**Figure 2:**
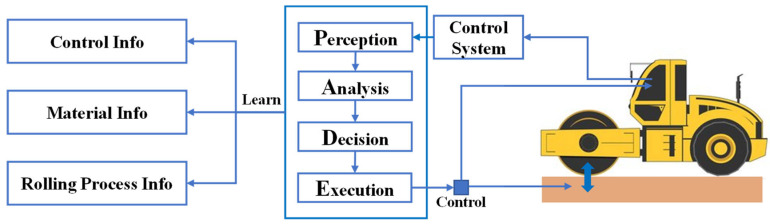
Information supervision system of IC.

IC technology lies in the meticulous monitoring of compaction machinery through a comprehensive information supervision system. This system utilizes a self-established positioning reference base station to deliver position differential signals to the mobile station installed on the compaction machinery, thereby achieving positioning accuracy at the centimeter level. Moreover, the compaction quality is enhanced by evaluating the material’s compaction state using parameters such as rolling speed, rolling temperature, rolling times, and compaction degree detection value [42,43]. Figure 2 presents the fundamental principle of the IC information supervision system.

**Figure 3:**
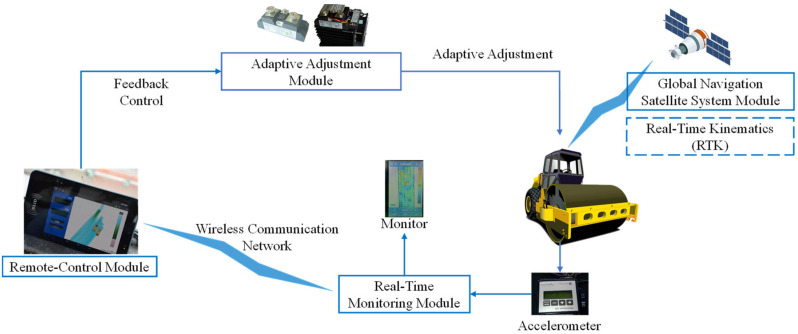
IC technology architecture.

To establish an integrated management mechanism for information sharing and collaboration among operators, contractors, supervisors, and owners, IC technology adopts the architecture depicted in Figure 3. This designed system streamlines the workflow and ensures optimal work efficiency, while simultaneously meeting the stringent quality control requirements of construction projects [13].

**Figure 4:**
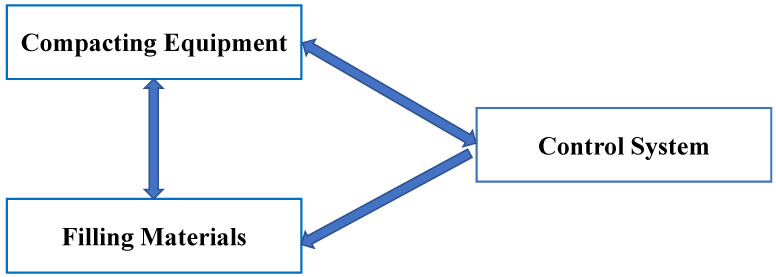
Interaction of filling body–roller–control system.

The concept of IC encompasses a broad spectrum of components and functionalities. While compacting machines and tools like road rollers and pavers are central to the process, IC extends beyond them. Even equipment without autonomous control over-compaction parameters can participate in IC. However, the key driving force behind IC is the advanced control system. This system enables the transmission of precise feedback control instructions to operators and facilitates automated regulation and control of compacting machines and tools. The intelligence exhibited by the control system is crucial for the success of IC. It enables the harmonious fusion and comprehensive performance resulting from the interaction among the filling body, compaction tool, and control system, as depicted in Figure 4. The control system’s ability to adapt and optimize compaction parameters in real time is of utmost importance. It ensures efficient compaction processes and helps achieve the desired level of compaction uniformity and density [34].

### 2.3. Main Technical Characteristics of Intelligent Compaction

**Figure 5:**
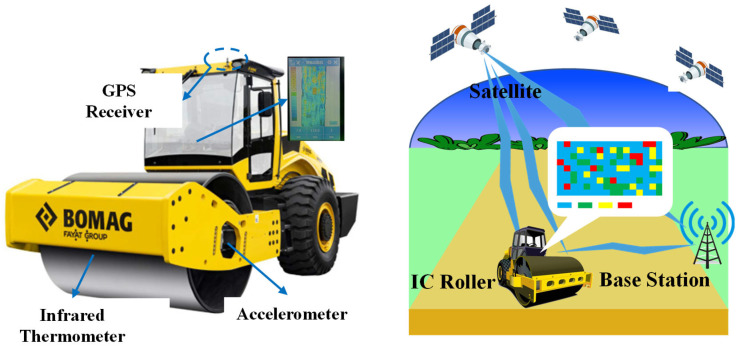
Schematic diagram of IC technology.

IC is distinguished by several key technical features. The vibratory roller is equipped with an accelerometer and infrared temperature sensor on the drum, while high-precision global positioning system equipment is utilized to map position data, which is then displayed to the roller operator in real time on the vehicle [44]. The measurement results of IC are presented as a color-coded map (Red indicates Compaction Measurement Value (CMV) below 20, yellow indicates CMV between 20–40, green indicates CMV between 40–80, and blue indicates CMV above 80) as depicted in Figure 5.

#### 2.3.1. Comparison between IC and Traditional Compaction Technology

In contrast to conventional compaction methods, the IC technology for pavement construction demonstrates notable distinctions in terms of intelligent control, data recording, and construction cost, as indicated in Table 2. IC technology is significantly superior to traditional techniques in ensuring compaction quality and record management, and it can provide more accurate and timely data support. The main advantage of this technology is its real-time control capability throughout the entire process, including more precise layer thickness control and dynamic data monitoring, which help improve road quality and extend road life. However, when ultimately deciding which compaction technology to use, comprehensive considerations must be made based on the specific needs, budget, and long-term maintenance costs of the project.

#### 2.3.2. Main Technical Features

IC is well-suited for a variety of materials, ensuring optimal compaction during construction—prominently for non-cohesive granular soils, fine-grained soils, crushed base materials, and asphalt mixes. IC offers the following advantages [33,45,46,47]:

(1) Enhanced control and accuracy: IC systems allow operators to monitor real-time compaction from the cab’s control box, enhancing visibility and reducing human error. This leads to fewer instances of under or over-compaction. Additionally, data records enable precise identification and rework of weakly compacted areas, ensuring a uniform compaction quality.
(2) Long-term benefits: Materials exhibiting low variability contribute to improved pavement performance and reduced maintenance costs in the future. The IC detection technology enhances compactness, flatness, and compaction uniformity of the subgrade and pavement, while also closely monitoring material rigidity during re-compaction, thus mitigating material variability.
(3) Holistic data utilization: IC systems, equipped with a suite of sensors, gather numerous quality metrics throughout compaction, facilitating immediate operative adjustments and long-term digital records. These parameters are valuable not only for real-time operator utilization but also for digital storage, serving as a foundation for road operation and maintenance in the subsequent life cycle.
(4) Enhanced construction quality and efficiency: The measured data parameters from IC can significantly improve road construction quality. The system also enables process control during compaction, resulting in reduced construction costs and improved efficiency. The luffing control system automatically adjusts the amplitude based on material compactness variations during compaction, thereby achieving a more even, rapid, and thorough pavement material compaction.
(5) Optimized compaction energy: The roller can automatically adjust the vibration compaction energy in accordance with soil compactness. This allows the pavement material to be compacted efficiently with fewer compaction cycles.

### 2.4. Process and Key Technology of Intelligent Compaction

The IC technology, as one of the core components in the construction phase of filling engineering, is experiencing rapid development and is considered the most mature technology in this field. The process of IC is not complex due to the automation of the machines involved. This approach aligns with the principle that the more intelligent a system becomes, the simpler its operation should be. However, the underlying technology behind IC is intricate and encompasses various disciplines [15,33,43,48,49,50,51,52].

**Figure 6:**
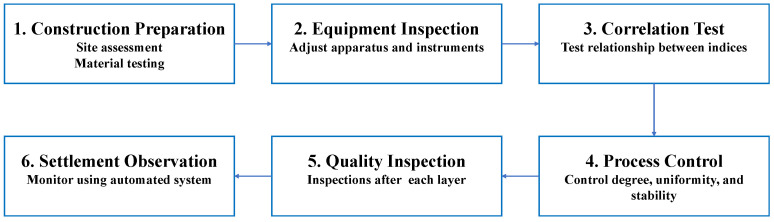
IC procedure.

The IC process involves six stages (Figure 6): construction preparation, equipment inspection, correlation testing, process control, quality inspection, and settlement observation [48].

(1) Construction preparation

Perform an exhaustive assessment of the site to pinpoint prospective challenges including the intricacies of the landscape, the properties of the soil, the presence of subsurface infrastructure, and additional unique obstacles pertinent to the location. Additionally, material testing should be conducted to ensure compliance with specifications and standards. This includes testing the aggregate gradation, asphalt binder properties, and mixture proportions to optimize the asphalt mixture for IC.

(2) Equipment inspection

Ascertain that the apparatus employed for densification is precisely adjusted, incorporating instrumentation such as accelerometers and navigational frameworks to meticulously oversee the compaction process. Fine-tune the apparatus to conform to the distinctive vibratory frequency and amplitude prerequisites of the asphalt amalgam, and authenticate the veracity of all gauging implements to guarantee the exactitude of the collected data. The compaction equipment should also be adjusted to the appropriate vibration frequency and amplitude for the specific asphalt mixture. Thorough inspections of the loading and measuring equipment should be conducted to ensure data authenticity and accuracy.

(3) Correlation test

This test establishes the relationship between the compaction control index and traditional quality indices. Through the densification of distinct test segments to varying degrees, quantifications are recorded to establish a concordance with the pre-established norms of excellence. Should the coefficient of correlation (r) attain or surpass the value of 0.7, such concordance is considered adequate for the establishment of compaction objectives.

(4) Process control

During the compaction process, it is crucial to control the compaction degree, uniformity, and stability. Communication technology is utilized to control the number of rolling times and the rolling track. The IC curve, recorded during the compaction process, is commonly used to evaluate the uniformity of subgrade compaction.

(5) Quality inspection

Self-inspection should be conducted after completing each layer, and inspections should be carried out once the construction meets the required standards. This process should be conducted layer by layer, section by section, and segment by segment.

(6) Settlement observation

The automatic settlement monitoring system is an unattended automated system that utilizes embedded sensors, data acquisition systems, transmission systems, and a control center to monitor settlement. Regular management and analysis of the collected data are performed within this system to track and assess settlement patterns.

In summary, IC optimally leverages sensing, computation, communications and control through its distinguishing properties. Continuous feedback control yields more consistent outcomes versus batch-style verification. However, while progress has been substantial, further validation under varied real-world conditions remains essential to unlock IC’s full potential [53,54,55]. The following findings can be drawn:

(1) IC technologies have progressed considerably in recent decades through innovations in sensing, data transmission, and control automation. At the core of IC systems lies the continuous acquisition of quantitative metrics capturing a pavement material’s compaction response through strategically positioned instruments like accelerometers, pressure sensors, thermometers, and GNSS receivers [56].
(2) Mechanically, modern IC rollers are equipped with vibratory drums, adjustable oscillators and automatic steering to uniformly impart controlled pressures [31]. At the helm of autonomous operations are intelligent control modules running real-time operating systems alongside specialized microcontrollers and high-performance computing resources. Accurate sub-decimeter positioning across job sites is enabled by integrated GNSS/IMU solutions along with range finding sensors. These core hardware and software constituents are networked through short-/long-range wireless and satellite links to facilitate distributed edge/cloud processing and visualization.
(3) Distinguishing IC from manual methods is its abilities to continuously monitor compaction in real time, directly feeding back diagnostics to operators [57]. Servo drives automatically regulate excitation levels according to current stiffness readings inferred from sophisticated measurement systems. Pervasive spatial coverage furnishes location-tagged data across entire construction extents, identifying weak zones for remediation.

## 3. Application of Advanced Sensors in Intelligent Compaction

**Figure 7:**
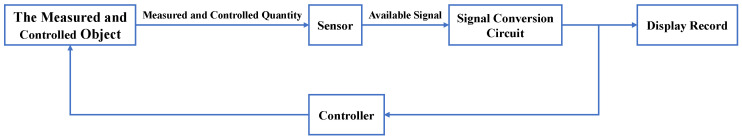
Automatic measurement and control system.

**Figure 8:**
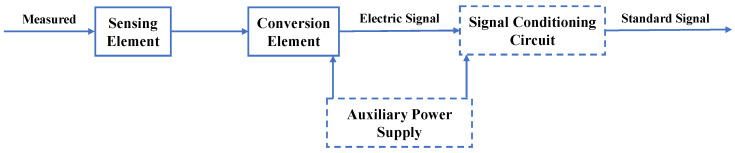
Block diagram of sensor composition.

Smart sensors play a crucial role in acquiring and transmitting information, making them essential tools in modern industrial processes, as shown in Figure 7. A sensor is a device that can detect and measure specific physical quantities and convert them into usable output signals according to a defined rule [58]. It typically consists of a sensitive element and a conversion element, as illustrated in Figure 8. The widespread use of sensors can be observed in various fields, including industry, agriculture, transportation, aerospace, national defense, resource exploration, marine development, environmental monitoring, security protection, medical diagnosis, bioengineering, household appliances, and more. In IC, the evolution of sensor technology is inextricably linked with the forward march of innovation. The IC system relies on the installation of various sensors on road rollers, along with microwave communication antennas, satellite positioning, and other technologies. Through wireless digital transmission via the internet, the system enables visual monitoring of subgrade compaction quality by project owners, supervisors, and construction personnel on computer and mobile devices.

By utilizing sensors, the IC system can achieve accurate positioning of road rollers, collect real-time data on compaction parameters, and provide valuable feedback to operators. The sensors measure and analyze factors such as vibration, temperature, and compaction effort, allowing for precise control of the compaction process. The continuous development of sensor technology, along with advancements in communication and data processing, has greatly contributed to the progress of IC.

### 3.1. Technical Principle of Sensors

**Figure 9:**
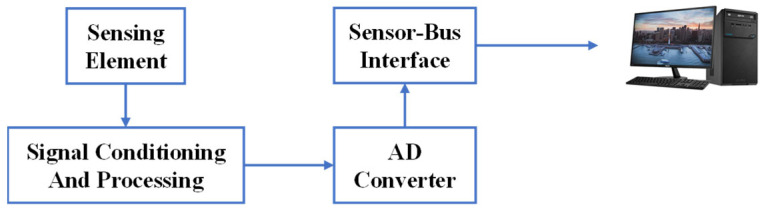
Components and principles of conventional sensors.

**Figure 10:**
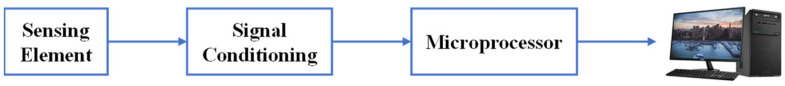
Components and principles of smart sensors.

The purpose of sensors is to convert the measured physical quantities into output signals (such as voltage, current, light wavelength, etc.) that can be utilized for information transmission, processing, storage, display, recording, and control. Sensors provide fundamental and intuitive signals during the compaction process, serving as a crucial hardware component for the entire IC system. Figure 9 illustrates a conventional sensor, which can be divided into four parts: (1) sensing element, (2) signal conditioning and processing equipment, (3) signal processing equipment, and (4) A/D converter and sensor interface [55]. The key distinction between smart sensors and traditional sensors lies in their intelligent capabilities, specifically the inclusion of an onboard microprocessor [59]. Figure 10 illustrates the components and principles of smart sensors.

### 3.2. Advanced Sensors in Intelligent Compaction

In the field of IC, several advanced sensors are commonly used to enhance the monitoring and control of the compaction process. These sensors include SmartRock sensors, FBG sensors, and integrated circuits piezoelectric (ICP) acceleration sensors [51,60,61,62,63].

#### 3.2.1. SmartRock Sensors

**Figure 11:**
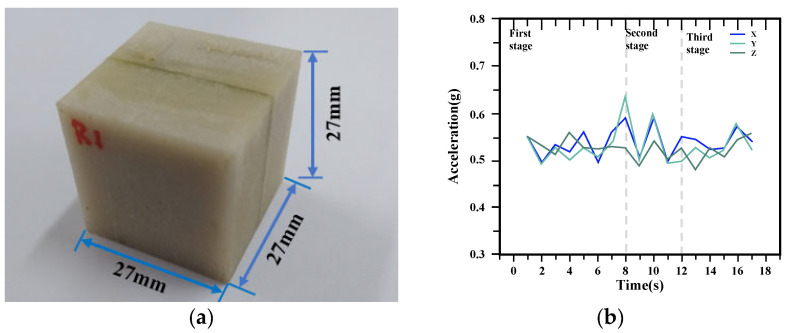
SmartRock System: (**a**) appearance (**b**) SmartRock received data. (The gray vertical dotted line is the boundary line for the monitoring stage).

SmartRock sensors are compact devices, typically 27 mm in size, that are fully embedded in a cubic enclosure [64,65]. Figure 11 illustrates its specific form and the received data. These sensors utilize various sensing elements such as gyroscopes, accelerometers, magnetometers, and stress meters to collect real-time data on parameters like time, acceleration, quaternion, and temperature. The collected data is wirelessly transmitted in real time using low-power Bluetooth technology and can be uploaded to cloud storage [66]. SmartRock sensors are designed to withstand high temperatures up to 150 °C, making them suitable for use in high-temperature compaction environments [67,68].

SmartRock sensors have unique capabilities in studying particle movement and kinematics of basic materials [64,68]. Through built-in acceleration sensors, temperature sensors, and Bluetooth modules, SmartRock sensors can collect real-time and wireless data related to stress, vibration acceleration, and rotation angle of asphalt pavement under external loads, quantitatively analyze particle migration and motion during the vibration compaction process of graded crushed stones, and contribute to the study of vibration compaction from macro to micro mechanical levels. Originally used for monitoring the stability of railway ballast, SmartRock sensors have also found applications in studying asphalt mixtures [46,69,70]. Their high integration, functionality, durability, stability, and ease of use make them advantageous compared to many traditional sensors used in asphalt pavements [60].

#### 3.2.2. Fiber Bragg Grating Sensors

FBG sensors have gained popularity in the evaluation of asphalt mixture performance and monitoring its reaction [71,72]. FBG sensors are a type of sensor that rapidly developed in recent decades. They offer advantages such as resistance to electromagnetic interference, good electrical insulation, high sensitivity, embed ability, and high frequency response. These sensors are widely used in health monitoring of critical infrastructure projects and have significant application potential [73,74].

**Figure 12:**
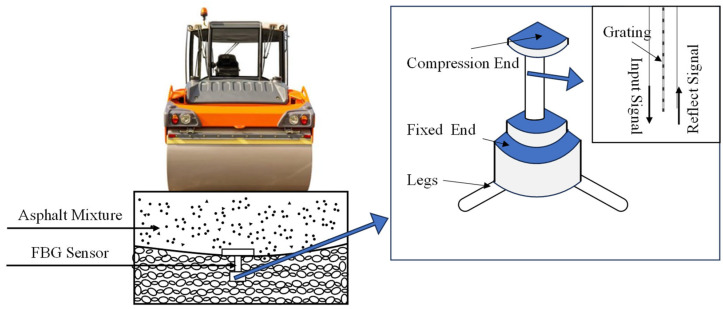
FBG sensor embedded in asphalt pavement.

Fiber optics undergo deformation under external forces, temperatures, and other loads, which can cause changes in the optical characteristics of the light transmitted within the fiber, such as intensity, wavelength, polarization state, etc. [75]. By using detectors such as spectroscopic analysis to measure the changes in these optical characteristics, the deformation of the optical fiber can be calculated based on its corresponding relationship with deformation, and then the load on the optical fiber can be calculated. The FBG sensors operate based on the principle of measuring changes in strain and temperature by monitoring the shift in the Bragg reflection center wavelength [76,77,78]. They typically consist of four parts: the compression end, detection end, fixed end, and legs, as shown in Figure 12. Different FBG sensors may exhibit variations in sensing sensitivity due to differences in fiber and grating writing techniques, as well as fabrication errors. Calibration is necessary before using FBG sensors for measurements, as materials, packaging technology, and temperature can affect their sensing performance [79].

#### 3.2.3. Integrated Circuits Piezoelectric Acceleration Sensor

ICP accelerometers are widely used in various vibration testing applications due to their small size, low susceptibility to interference, high sensitivity, cost-effectiveness, and ease of installation. The output of the ICP accelerometer is a voltage signal, so the size of the collected voltage signal indirectly reflects the changes in the vibration acceleration of the vibrating wheel. The two are in a linear relationship. After analyzing the data through self-spectrum (power spectrum) analysis, it was found that as the number of compaction passes increases, the ratio of the second harmonic amplitude to the fundamental amplitude gradually increases, and the compaction degree also gradually increases. In this way, the variation of this ratio can be compared with the compaction degree measured by the sand filling method, and a database can be established to form a built-in expert system. Then, direct reading of the compaction degree value can be achieved through software programming [80].

In different application scenarios, ICP acceleration sensors, FBG sensors, and SmartRock sensors have their own unique advantages and disadvantages. Therefore, when choosing the right sensor, it is necessary to make a comprehensive consideration based on the specific application needs and budget. The strengths and weaknesses of the three different sensors are summarized in Table 3.

### 3.3. Application of Sensors in Intelligent Compaction

For IC systems, the task of accurately collecting relevant data information is mainly achieved through sensor devices installed in the roller equipment. During the compaction operation, the amplitude on the rolling wheel will undergo significant changes due to the influence of changes in the compaction degree of the road surface material. By scientifically analyzing key features, compaction detection values can be generated. Then these values can be compared with the expected compaction degree to determine the state of the compacted material.

Yu and Shen [67] conducted a study using SmartRock sensors (STRDAL Intelligent Technology Co., LTD, Nanjing, China), a wireless particle size sensor, to investigate the compaction performance of asphalt mixtures. They embedded SmartRock sensors in eleven asphalt mixtures and monitored the compaction process. This study aimed to understand the compaction characteristics of particles in relation to the overall compaction performance.

**Figure 13:**
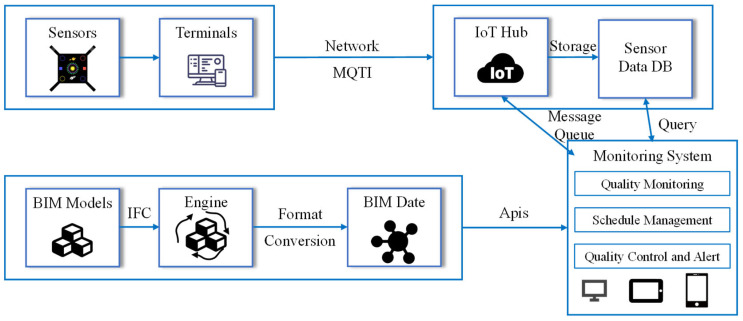
Intelligent compaction system with BIM–IoT technology.

Han et al. [51] developed an IC technology system based on BIM and Internet of Things (IoT) technology. They created a prototype system for real-time monitoring and management of the compaction process, as shown in Figure 13. The system utilizes high-precision sensors such as acceleration sensors, speed sensors, and temperature sensors to collect construction data during the compaction process.

Tan et al. [81] proposed the application framework of FBG sensing technology in quality control for asphalt pavement compaction. They embedded FBG sensors in asphalt pavements to identify areas with weak compaction based on the response values of the sensors. Additionally, the FBG sensors can be used for long-term monitoring of pavement structural performance.

Wang, et al. [82] utilized the function that SmartRock sensors can continuously monitor the stress, rotation, and acceleration changes of aggregates under the action of gyratory compaction to evaluate and monitor the compaction status of asphalt mixtures in real time. It provided a solution to optimize the compaction process and improve the quality of construction.

Tang et al. [63] designed and developed an impact construction acceleration information collection device based on ICP acceleration sensors and satellite positioning measurement technology. They conducted compaction analysis on road rollers, determined compaction quality, and promptly reworked unsatisfactory road sections to ensure construction quality.

In 2020, the Zoomlion ZRS322E single drum IC roller, used in the construction of a cement stabilized macadam base road in Shandong Province, was equipped with an acceleration sensor [56]. This sensor collected data on the vibration acceleration in the vertical direction of the vibrating wheel, enabling the assessment of the subgrade compaction degree.

These examples demonstrate the application of advanced sensors such as SmartRock, FBG sensors, and acceleration sensors in the field of IC. These sensors provide valuable data for monitoring and controlling the compaction process, improving the efficiency and quality of compaction operations.

Accurate sensing lies at the core of IC systems, and recent innovations have aimed to enhance IC monitoring capabilities. SmartRock sensors, FBG sensors, and ICP acceleration sensors are several commonly used advanced sensors in the field of IC, each with its unique advantages and application scenarios. The following findings can be drawn:

(1) SmartRocks are compact, durable devices that incorporate MEMS gyroscopes, accelerometers, and Bluetooth to wirelessly transmit real-time data on stress, rotation, and acceleration from within mixtures under compaction. This allows quantitative analysis of particle migration mechanics. FBG sensors embed fiber optics to detect strain and temperature changes through Bragg wavelength shifts, finding use in pavement response and performance monitoring. ICP sensors are well-suited for vibration measurement applications given their small size, stability, and cost-effectiveness.
(2) Each sensor type presents unique strengths—SmartRocks probe internal material behavior while FBG and ICP devices assess external responses. However, protecting sensors from jobsite hazards and maintaining calibration stability over the long run remain challenges.
(3) In practice, most sensors are installed on rollers to collect pressure, vibration, and thermal parameters through positioning systems, guiding compaction control, and quality evaluation. Recent studies explored utilizing SmartRocks to investigate mixture compaction characteristics and FBG networks to localize weak zones requiring remediation.

Overall, advanced sensing is pivotal for IC, yet further improvements in robustness under harsh conditions are still needed. Future research must also focus on validating sensing techniques using extensive field data to ensure reliability under diverse scenarios. Their integration into larger digital construction frameworks also shows potential.

## 4. Intelligent Compaction Evaluation

In recent years, significant progress has been made in the field of IC, particularly in the context of highway construction. Researchers have dedicated their efforts to developing techniques and technologies that enhance the compaction process and ensure improved compaction quality. This focus is crucial because compaction plays a vital role in the long-term durability and performance of road pavements. As modern control technology progresses, vibratory compaction parameters can now be automatically fine-tuned. Nevertheless, identifying the suitable adjustments and their optimal values poses a persistent challenge. Extensive vibration compaction test findings indicate that distinct fillers, or even the same filler with varying gradations, necessitate specific combinations of vibration compaction process parameters (including exciting force, vibration quality, vibration frequency, amplitude, etc.) to attain the most effective compaction outcome.

To evaluate the compaction quality of the subgrade in real time and meet specified requirements, various compaction quality evaluation indices are utilized. These indices include the CMV, Compaction Control Value (CCV), Mechanical Driving Power (MDP), Accelerated Intelligent Compaction Value (AICV), Intelligent Compaction Measurement Value (ICMV), Vibration Compaction Value (VCV), acceleration amplitude, and other relevant metrics [26,33,83,84,85,86,87,88]. In particular, CMV is widely employed to assess the stiffness of the compacted material during the compaction process.

Furthermore, Liu et al. [89] introduced the concept of the compaction value to represent the overall compaction effect of the subgrade. Building upon this concept, they established a rapid evaluation method for assessing the compaction quality of the entire construction site based on the compaction value. This approach enables a comprehensive evaluation of compaction quality and facilitates timely adjustments to the compaction process to meet specific requirements.

(1) Compaction Measurement Value

The CMV is determined by considering the mechanical interaction between the vibrating drum and the compacted layer, providing an indication of the soil’s stiffness to a certain extent. To calculate the CMV, the vertical acceleration time spectrum signal is collected using an acceleration sensor during the compaction process. This signal is then subjected to a discrete Fourier transform (DFT) to obtain the frequency spectrum signal of the vibration acceleration. The frequency spectrum signal represents the distribution of frequencies and their corresponding magnitudes within the vibration acceleration. By analyzing this signal, the CMV is derived, serving as a valuable metric for assessing the compaction quality of the material being compacted. It is important to note that this calculation method for CMV is just one of several techniques employed in IC to evaluate compaction quality, and ongoing research aims to enhance the accuracy and efficiency of compaction assessment methods. The calculation equation is shown in Equation (1) [90].

(1) $$CMV=C\frac{A_{1}}{A_{0}}$$

where *C* is a constant, and the vibration frequency, amplitude, type of vibratory roller, and other factors shall be selected according to the actual situation; *A*_1_ is the acceleration amplitude of the first-order harmonic component of vibration; and *A*_0_ is the acceleration amplitude of the basic component of vibration.

(2) Compaction Control Value

The CCV is a relative index that reflects the stiffness of the material being compacted and serves as an enhanced version of the CMV by considering subharmonic components. The CCV takes into account the Compaction Control Value of the underlying basement layer, exhibiting a consistent change trend with it. The calculation of the CCV involves the utilization of acceleration data for the first harmonic, fundamental, and higher harmonic amplitudes, as shown in Equation (2) [91].

(2) $$CCV=100\frac{A_{0.5\Omega }+A_{1.5\Omega }+A_{2\Omega }+A_{2.5\Omega }+A_{3\Omega }}{A_{\Omega }+A_{0.5\Omega }}$$

where *A*_3Ω_ is the acceleration amplitude of the second harmonic component, *A*_Ω_ is the first harmonic corresponding to the amplitude, A_0.5Ω_, *A*_1.5Ω_, *A*_2.5Ω_, and *A*_3.5Ω_ are the acceleration amplitudes of the fundamental frequencies of 0.5, 1.5, 2.5, and 3.5, respectively.

(3) Mechanical Driving Power

The MDP is a significant indicator used to assess the compaction quality of materials. It measures the power required by the compactor’s drive system to achieve the desired compaction level. The MDP is calculated by monitoring the power consumption of the compactor during the compaction process. However, the current evaluation of this index lacks rigorous theoretical derivation and relies on empirical estimates.

The MDP serves as a valuable metric for evaluating the compaction process and ensuring the desired compaction level is achieved. It can be used to optimize compaction operations by adjusting the compactor’s parameters, such as frequency and amplitude, to achieve the desired compaction energy. Monitoring the MDP during compaction allows for real-time assessment of the compaction quality and enables adjustments to be made if necessary to achieve the desired compaction specifications.

(4) Accelerated Intelligent Compaction Value

The AICV is a calculation that focuses specifically on the third harmonic during the compaction process. It is used as an index to evaluate the compaction quality of materials, as shown in Equation (3) [47]. The AICV provides valuable information about the compaction quality by specifically considering the contribution of the third harmonic. This harmonic is associated with certain characteristics of the material’s response to compaction forces. By focusing on the third harmonic, the AICV offers a more targeted assessment of the material’s compaction behavior.

(3) $$AICV=C\frac{A_{2\Omega }+A_{3\Omega }}{A_{\Omega }}$$

where *C* is a constant, 100, Δ_2Ω_ is the amplitude corresponding to the second harmonic, Δ_Ω_ is the first harmonic corresponding to the amplitude, and Δ_3Ω_ is the amplitude of the third harmonic.

Monitoring the AICV during the compaction process allows for real-time evaluation of the compaction quality and enables adjustments to be made if necessary. It serves as a useful tool for optimizing compaction operations and ensuring that the desired compaction specifications are met.

Compaction quality evaluation indices have the advantage of objectivity and simplicity in evaluating problems, which can facilitate the comparison of different systems or methods. However, we should also be aware of its limitations, and it is necessary to consider multiple indicators in a comprehensive way and to select appropriate evaluation metrics on a case-by-case basis to obtain more comprehensive and accurate results. Table 4 summarizes the advantages and disadvantages of different compaction quality evaluation indices.

IC evaluation indexes play an important role in quantifying compaction quality in real time. To assess the quality of subgrade compaction in real time, researchers utilize various evaluation indices including the CMV, CCV, MDP, AICV, and ICMV. The following findings can be drawn:

(1) The CMV utilizes acceleration signals derived from vibratory drum motions using Fourier transform analysis. The CCV builds upon the CMV by incorporating additional subharmonic components. The MDP gauges the mechanical work input required by compactors, though its theoretical basis requires further development. The AICV focuses analysis on the critical third harmonic component. The ICMV integrates data from multiple sensors into a standardized metric.
(2) While these metrics provide an objective means for evaluation, each indicator presents limitations that may be overcome by using multiple metrics comprehensively. For example, CMV/CCV/AICV metrics require establishing baseline correlations, and discrete test samples may not fully represent whole construction areas.
(3) Future work should continue refining theoretical underpinnings and validation of evaluation methods considering project-specific conditions. Overall, IC evaluation indexes streamline quality control but balancing individual strengths and weaknesses remains important.

## 5. Machine Learning in Intelligent Compaction

ML plays a significant role in IC evaluation for asphalt pavements. It serves as the core of data analysis algorithms, which process and analyze sensor data using techniques like ML, statistics, and model prediction to assess compaction quality. By applying ML methods, the accuracy of compaction prediction can be enhanced, leading to optimized construction processes and improved quality control. The ML algorithms are capable of constructing intricate models that learn from and analyze extensive datasets to predict compaction quality.

In comparison to traditional empirical formulas and rules, ML models offer more precise predictions of the properties and behavior of soil or rock materials under various compaction conditions. Additionally, these models can detect potential quality issues and risks at an early stage, enabling construction workers to take prompt measures to prevent accidents and ensure quality standards.

### 5.1. Machine Learning

**Figure 14:**
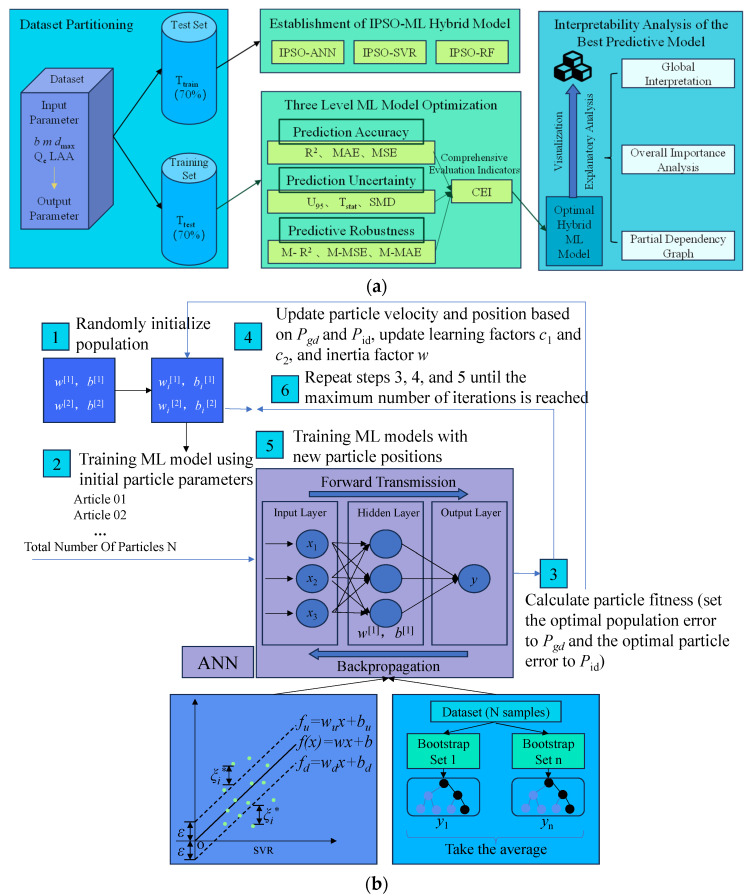
ML-based prediction model framework. (**a**) ML-based framework for predicting embedded locking points; (**b**) Optimization process of PSO–ML hybrid model. (*w*^1^ and *b*^1^ represent the input speed and position, while *w*^2^ and *b*^2^ represent the output speed and position, *ξ*_i_* represents the dispersion between real data and linear fitting).

ML has emerged as a powerful tool for enhancing data processing efficiency. In the field of IC, researchers have utilized ML techniques, particularly artificial neural network models, to address geotechnical engineering challenges [92,93]. For instance, Isik and Ozden [94] developed an artificial neural network model to estimate compaction parameters for a wide range of soil mixtures, demonstrating the applicability and effectiveness of their approach. In recent years, ML algorithms have found extensive applications in various areas of pavement engineering, including distress classification, performance prediction, and dynamic modulus back-calculation [95,96]. Leveraging sensor data in conjunction with ML technology has proven to be an effective method for predicting the compaction state of asphalt mixtures [67]. As shown in Figure 14, the prediction model framework based on ML highlights its powerful functionality and prospects in practical operations in this application field.

However, future research in this field should focus on evaluating the algorithms using field compaction data to ensure their practicality and reliability. Additionally, efforts should be made to advance intelligent sensing technology, enabling real-time monitoring and feedback during the compaction process. This would facilitate continuous improvement in compaction quality and enhance the overall performance of asphalt pavements.

### 5.2. Common Machine Learning Algorithms

ML emerged as a scientific topic in the 1980s after being proposed in the 1950s [97]. Reasoning, knowledge, formation, and wealth have characterized its development. There are many ML classification methods. Output results classify it as classification or regression. Supervised, semi-supervised, and unsupervised learning depend on whether the training dataset needs annotation. The amount of tasks handled determines single-task or multi-task learning.

#### 5.2.1. Regression Model

Common regression models include multiple linear regression (MLR), ridge regression (RR), and Lasso regression (LR). Below is a brief introduction to each model.

(1) MLR is a type of linear regression model that has a larger number of features compared to traditional linear regression [98]. Its general form is

(4) $$Y=\beta_{0}+\beta_{1}X_{1}+\beta_{2}X_{2}+\ldots +\beta_{n}X_{n}$$

where *Y* is the output variable; *X*_i_ is the corresponding variable; and *β*_i_ is the slope coefficient of the corresponding variable. MLR is commonly used in linear multivariate analysis, and the factors are independent of each other.

(2) RR is an improved least squares algorithm. When the regularization factor is chosen as the binomial of the model parameters, the regression method is called RR [99], which can solve the problem of data features being larger than the number of samples. The solution of ridge regression is

(5) $$\omega ={arg}_{\omega }^{min}({\left\|y-X_{\omega }\right\|}_{2}^{2}+\lambda {\left\|\omega \right\|}_{2}^{2})$$

where *X* is the input feature matrix; *Y* is the output matrix; *ω* is the parameter vector of the model; and *λ* is the ridge regression parameters.

(3) LR is a type of compressed estimation. By adding L1 norm penalty as a penalty function to the loss function, a more refined model is obtained that can compress some coefficients to zero [100]. The loss function formula is

(6) $$\arg\underset{\beta }{\min}\frac{1}{N}\sum_{N}^{i=1}{\left(y_{i}-\sum_{m}^{p=1}\beta_{p}x_{p}^{n}\right)}^{2}+\lambda \sum_{m}^{p=1}|\alpha_{p}|$$

where *β* is the parameter vector to be optimized; *y*_i_ is the dependent variable value of sample i; *N* is the number of samples; *M* is the number of feature parameters; $x_{p}^{n}$ is the p-th feature of the nth sample; *α*_P_ is the regression coefficient of the p-th feature; and *λ* is the hyper parameters. LR has the advantage of subset contraction and can handle data with multicollinearity, which refers to data with a greater number of sample features than the number of samples.

#### 5.2.2. Classification Model

Classification models play a significant role in ML algorithms and have a greater number of practical applications compared to regression models. Typical classification models comprise decision trees (DTs), Bayesian classifiers, support vector machines (SVMs), and ensemble learning.

(1) A DT is a model approach in which non-leaf nodes represent feature attributes, branch edges reflect the output of the feature attribute within a specific value range, and leaf nodes represent the final category [101]. The classification path is the route from the root node to the leaf node, and only samples that satisfy the different criteria along the classification path will be assigned to that category.
(2) A Bayesian classifier is a technique for classifying data based on the Bayesian theorem and the assumption that attribute features are independent. It calculates the likelihood of a sample belonging to a specific class [102]. The Bayesian classifiers algorithm is characterized by its straightforward algorithm logic and ease of implementation, distinguishing it from other classification algorithms. The classification method incurs a reduced spatial overhead. Nevertheless, in real-world scenarios, achieving perfect independence across all attributes is challenging, leading to subpar classification performance of the Bayesian approach.
(3) A SVM is a supervised binary classification model that uses a sum function to train and construct a hyperplane [103]. This hyperplane allows the SVM to accurately classify most samples in the sample set and maximize the distance between the samples and the hyperplane. As a result, the SVM achieves strong generalization ability.
(4) Ensemble learning refers to a strategy of constructing multiple learners to complete classification tasks, which can combine the advantages of each learner and improve classification performance. Common ensemble learning methods include Boosting, Bagging, and Stacking [104]. Boosting focuses more on the portion of samples with training errors during training, and constructs a new classifier by strengthening attention to errors to improve classification performance. Bagging constructs a classifier structure through random resampling of data and uses the bootstrap method to obtain N datasets from the overall sample set through put back sampling. Basic learners are trained separately on each dataset, and the final prediction result is obtained by voting on the output of N models. Stacking is a type of stacked ensemble model, in which the training results of each layer will be used as input for the next layer, and ultimately the final layer’s learner will perform comprehensive training as the output of the model.

#### 5.2.3. Artificial Neural Network

An artificial neural network (ANN) imitates biological neural networks for function fitting and is the foundation of various popular neural networks [105]. It has strong parallel processing ability, fault tolerance, and automated intelligent learning ability. An ANN is composed of a large number of interconnected nodes, each representing an activation function, and the connections between nodes represent the weights passing through the path. The output result of a network is determined by the overall structure of the network, the connection method of network nodes, the activation function, and the weights.

According to different network structures or implementations, an ANN can derive various models, such as a Multi-Layer Perceptron, Extreme Learning Machine, Generalized Regression Neural Network, and Backpropagation Neural Network.

### 5.3. Application of ML in Intelligent Compaction

The combination of ML technology and IC technology can improve efficiency, ensure quality, and reduce resource waste in compaction operations of soil, asphalt, and other building materials. IC technology uses various sensors (such as accelerometers, GPS, pressure sensors, etc.) to collect data on the compaction process, such as the speed of compaction machinery, vibration frequency, rolling frequency, density and hardness of soil or materials, etc. These data can be used to monitor and optimize the compaction process. The ML models can assess the compactness and uniformity of the ground and provide real-time feedback on quality. By monitoring and analyzing compactor sensor data in real time, ML models can detect abnormal patterns and predict potential failures. This predictive maintenance approach helps reduce equipment downtime and maintenance costs, while improving the reliability and stability of construction projects. The application of ML in IC can be categorized into two main aspects: data acquisition and predictive modeling.

#### 5.3.1. Data Collection

Data acquisition serves as the foundation of IC. Through sensor networks and data acquisition devices, real-time collection of various parameters such as pressure, vibration, temperature, etc., from the soil and road can be achieved. This data is then transmitted to ML algorithms for analysis and processing. By learning from large datasets, ML can identify characteristic patterns of soil and roads and correlate them with successful compaction outcomes.

There are several common methods for data collection, including the following:

(1) Sensor Data: Various sensors, such as accelerometers, pressure sensors, wireless sensors, and displacement sensors, are used to monitor the state and behavior of the compactor during construction [51,67,106]. These sensors record information such as vibration, pressure, displacement, etc., providing valuable data for ML algorithms. However, significant practical operations concerns remain. First, sensor placement must be sensible to cover essential measurement locations and offer useful data. Meanwhile, in hard construction conditions, protecting sensors from damage and maintaining calibration to ensure long-term data correctness is a question. In addition, sensor data demands a lot of storage space, and processing it for ML algorithm analysis requires powerful computing power and advanced data analysis.
(2) Image and Video Data: Cameras or other vision sensors capture image and video data of the compactor and the construction site [107,108]. These data can be used to analyze the physical properties of the soil, the position and trajectory of the compactor, and other relevant information. The ML algorithms can extract features from images and videos to predict and control the compaction process. However, the data in images and videos not only requires complex preprocessing steps to ensure they can be effectively analyzed (such as filtering out noise, lighting changes, and other interfering factors), but they also require advanced methods for processing these data, such as computer vision and deep learning techniques.

Therefore, in order to optimize data quality and analysis accuracy, it is usually necessary to comprehensively apply multiple data sources and conduct intelligent analysis. The comprehensive use of these data collection methods can greatly enhance the learning effectiveness of ML algorithms and improve the intelligent monitoring and control level of compaction. However, this also presents new challenges, including how to efficiently integrate large amounts of data from multiple sources, and how to ensure the cost-effectiveness and sustainability of the data collection process. With the continuous development and optimization of technology, these challenges will gradually be overcome, providing stronger support for IC and precise engineering management.

#### 5.3.2. Predictive Modeling

ML can build predictive models that estimate the mechanical properties of soil and roads based on real-time collected data. These models can be adjusted according to different construction conditions and parameters to achieve optimal compaction effects. Predictive models provide real-time feedback and guidance to construction personnel, assisting them in making informed operational decisions [48].

Predictive modeling plays a crucial role in the field of IC by utilizing ML algorithms to analyze and interpret data, constructing models to predict the best practices for soil and road construction, and further improving construction quality and efficiency. However, in practical applications, predictive modeling is not just about building and running a model. It involves a series of complex steps, each of which is crucial and requires precise processing to ensure the successful application of the model.

(1) Data collection and preparation: This is the first step in modeling and a crucial part of the entire process [51]. Sufficient quantity and quality of data need to be collected to train and validate the model. In addition, data preparation not only needs to consider the diversity and comprehensiveness of the data, but also ensures the accuracy and completeness of the data. In the field of IC, this may mean collecting data from multiple sensors and data sources (including image and video data, sound data, sensors, etc.) and ensuring that these data can accurately reflect the actual situation of the construction site.
(2) Data preprocessing: The collected data often contains noise, missing values, or inconsistencies, which need to be cleaned and standardized through preprocessing steps [50]. This may include filtering out noise, filling in missing values, data normalization, etc., with the aim of making the data more suitable for model training and accurate prediction.
(3) Feature extraction and selection: This step involves extracting useful features from preprocessed data and selecting the most helpful features for the prediction model. Feature selection can not only improve the performance of the model, but also reduce the complexity and computation time of the model.
(4) Tagged data: Tagged data is necessary for supervised learning. This process involves assigning a correct output label to each sample in the dataset, so that the model can learn how to predict the performance of roads.
(5) Model selection and training: Choose a suitable ML model to train data, which may include regression models, neural networks, decision trees, etc. After selecting the model, use the training data to train the model until it can accurately predict or classify.
(6) Model evaluation and optimization: After the model training is completed, a series of evaluation indicators (such as accuracy, recall, F1 score, etc.) need to be used to test the performance of the model. Based on the evaluation results, it may be necessary to further adjust the model parameters for optimization to improve the accuracy of the prediction.
(7) Prediction and application: The evaluated and optimized model is ready to predict new data. The prediction results of the model can provide real-time feedback and guidance for construction personnel, helping them adjust construction strategies and achieve better compaction effects.

In summary, predictive modeling is a dynamic and iterative process that requires continuous feedback and optimization to adapt to new data and environmental changes. With the advancement of technology and the improvement of data collection technology, the accuracy and application scope of predictive models will continue to expand, providing stronger support for IC and construction management.

#### 5.3.3. Practical Applications of Machine Learning

Researchers have made practical advancements in combining ML methods with IC techniques. For example, Fathi et al. [109] combined ML methods with IC and modulus-based field testing to estimate the mechanical properties of compacted geotechnical materials. This approach served as a local calibration process. Pereira et al. [110] proposed using soil clay content, soil sand content, soil silt content, soil density, and soil volumetric moisture to predict soil firmness. They established prediction models using SVM and ANN, respectively, and compared them to find that SVM has a more stable comprehensive prediction performance. Wang et al. [111] developed a PSO–BP–NN model to predict the shear strength and compactness of subgrade soil within a range of mechanical properties and compaction force. This model improved the accuracy of IC by estimating compactness based on material properties.

Chen et al. [112] combined time domain and frequency domain characteristics with artificial neural networks to accurately evaluate overall soil compaction quality. This method effectively classified soil compaction quality into under-compaction, optimal compaction, and over-compaction. Xu et al. [93] established a numerical model to simulate the interaction between a vibrating drum and underground soil. The model measured soil stiffness in real time and estimated the degree of compaction, allowing for evaluation of construction effectiveness and adjustment of vibration parameters to achieve better compaction results. The model also provided predictions of land deformation and stress distribution, enabling construction personnel to identify potential quality problems and risks in a timely manner and take appropriate measures. Wang et al. [113] proposed a self-defined kernel support vector regression model for compaction quality evaluation based on an intelligent bacterial foraging algorithm. They adopted an enhanced probabilistic neural network for real-time compaction quality control. The model formulated effective feedback control measures and demonstrated its effectiveness and superiority in practical applications. These practical applications highlight the potential of ML in IC, paving the way for improved compaction quality control and construction efficiency.

ML has emerged as a powerful tool to enhance data-driven decision making in construction through intelligent analysis, pattern recognition, and predictive modeling. It revolutionizes the way compaction quality is assessed and controlled in IC applications. The following findings can be drawn:

(1) ML algorithms construct comprehensive models by training on vast amounts of historical sensor recordings and associated construction attributes. They can then generalize learnings to new scenarios, overcoming limitations of isolated experiments.
(2) Data collection serves as ML’s foundation, requiring representative, high-quality inputs. Section 5.3.1 reviewed common data sourcing techniques in IC like sensors, imagery, and acoustic signals. However, reliably obtaining data under field conditions presents difficulties from environmental influences, intensive work durations, and obstructions to discrete sensor deployments. Emerging wireless technologies may strengthen robustness and seamless integration of diverse modalities for well-rounded characterization.
(3) Multi-step procedures involving data preprocessing, feature selection, labeling, model selection/training, evaluation/optimization, and application in various domains were discussed. Standardization efforts could expedite sharing and cross-project learning. Simulations may supplement limited physical testing beds for model development and hypothesis validation.
(4) Regression, classification, and artificial neural network algorithms have proven useful in geotechnical scenarios, for instance, when estimating mechanical properties like strength and stiffness, evaluating compaction quality levels, and predicting moisture contents and densities based on material attributes. Hybridizing swarm optimization with deep networks has potential for further accuracy gains.
(5) Promising applications included estimating properties from field modulus tests, soil firmness prediction through support vector machines, shear strength/compactness forecasting using particle swarm-backpropagation neural networks, overall quality assessment integrating time/frequency domain learning, and numerical solvers simulating drum–soil interactions. Future work must perform exhaustive testing on pavement jobsites.

In summary, ML’s self-learning nature addresses gaps in empirical construction knowledge while massive online data resources fuel its potential. Notwithstanding ML’s demonstrated impacts, field-based research remains crucial before commercialization. Collaboration between experts from ML, geotechnical engineering and construction management will help maximally leverage data-driven technology for the IC domain. Concerted efforts validating diverse algorithms through live demonstrations can then establish ML-IC as a standardized precision construction solution.

## 6. Practical Application of Intelligent Compactor

The convergence of advanced sensing, computing, and control technologies has enabled the development of sophisticated IC machinery well suited for asphalt pavement and soil applications [114]. This section examined representative intelligent roller systems and case studies where IC yielded benefits.

### 6.1. Development and Application of Intelligent Rollers Abroad

IC technology originated in the 1970s, evolving through the 1980s as a sustainable construction practice, with Europe leading its development in the late 1970s. The first decade of the 21st century witnessed rapid development in intelligent rolling technology, with advancements in compaction control, precision, and automation by major manufacturers such as BOMAG, Caterpillar, and HAMM [15,27,115,116,117]. These innovations have contributed to improved compaction efficiency and quality in road construction projects. Table 5 and Table 6 summarize the main manufacturers and key technical parameters of intelligent road rollers for soil compaction and asphalt concrete pavement compaction.

### 6.2. Intelligent Compaction System

#### 6.2.1. BOMAG VARIOCONTROL (BVC)

The BVC system is an automated IC system that offers variable amplitude capabilities. It employs an advanced excitation system capable of generating precise directional vibrations, effectively transmitting the vibratory compaction force to the ground. By intelligently detecting the required vibration energy at different compaction stages, the system can dynamically adjust the vibratory compaction force in real time [118]. The BVC system demonstrates exceptional responsiveness, with adjustment times from vertical to horizontal orientations taking no longer than one second. This remarkable adaptability plays a crucial role in mitigating the potential risks associated with wheel jumping or tumbling caused by excessive compaction force. Furthermore, the user interface provides a graphical representation of the compaction zone, clearly distinguishing between standard and non-standard areas. The system allows for the recording of compaction zone values in the form of curves [83].

#### 6.2.2. AMMANN Compaction Expert (ACE)

The ACE system finds application in vibratory rollers, where it is employed for soil compaction purposes. Its function involves compacting paving materials until a specific stiffness threshold is attained. Once the desired stiffness level is reached, the roller transitions into a measurement mode, wherein it continues to assess the stiffness of pavement without engaging in further compaction. Notably, the continuous compaction control computer system is responsible for recording and storing both the pavement data and its corresponding location at the job site [45,119].

#### 6.2.3. DYNAPAC Single Drum Vibratory Roller Online Compaction Management System

The DYNAPAC compaction management system designed for single drum vibratory rollers offers multiple levels of configuration. The basic configuration includes a compactness meter, while the next level combines a compactness meter with a compactness analyzer. The most advanced configuration integrates a compactness meter, compactness analyzer, and GPS for comprehensive compaction management [14]. This system is capable of automatically adjusting the compaction parameters based on the condition of the compaction surface. It continuously measures the hardness of the compaction surface and alerts the operator to issues such as double jumping or over-compaction.

#### 6.2.4. Other Intelligent Compaction Technologies

HAMMTRONIC, developed by HAMM, is an IC control system that precisely manages the walking system and compaction vibration system of the equipment. It incorporates features such as constant speed cruise, constant vibration frequency, and automatic vibration start and stop. By reducing operator difficulties and minimizing errors, this system mitigates quality risks associated with human control.

Ingersoll Rand has introduced a compactness test system that includes a vibration step frequency meter on the instrument panel. This system assists operators in accurately controlling the ratio of vibration frequency to walking speed. It utilizes a compactness detection system to manage road surface compactness and enables real-time adjustment of roller working parameters based on the compactness data, thereby achieving optimal compaction and flatness.

These IC technologies, including BVC, AMMANN compaction expert, DYNAPAC compaction management system, HAMMTRONIC, and Ingersoll Rand compactness test system, offer advanced features and automation to enhance compaction efficiency and quality control in road construction projects.

The IC systems provide efficient and precise control, but they come with high investment and operating costs. Therefore, it is important to select the appropriate system based on project requirements and budget. Table 7 provides a summary of the advantages and disadvantages of different compaction systems.

### 6.3. Application Cases of Intelligent Compaction

**Figure 15:**
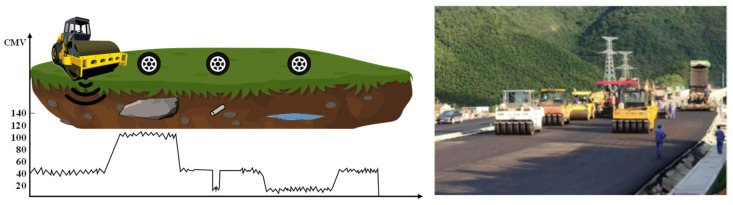
On-site testing of compaction degree.

IC technology has been effectively employed in numerous road construction projects, exemplifying its efficacy in enhancing the quality and efficiency of construction endeavors. As shown in Figure 15, the intelligent system can continuously measure the hardness of the compacted surface, warning the operator of double jumping or over-compaction. For instance, the Nanning–Zhanjiang Expressway project employed the Vogele Super 2100-3L paver in conjunction with Hamm HD series rollers, resulting in improved construction quality through the utilization of the BOMAG compaction measurement and documentation system. Similarly, the New Cologne–Rhine/Main Line project utilized a BW219PDH-3 pedal roller (BOMAG, Boppard, Germany) for double pressing, coupled with a BW219DH-3 smooth oscillating roller (BOMAG, Boppard, Germany), and reaped the benefits of IC technology [120]. In the TH14 Project, Ammann and Caterpillar rollers, equipped with the ACE measurement system, automatically adjusted the compaction energy based on in-situ soil stiffness measurements, leading to enhanced compaction efficiency [121]. Furthermore, the BVC intelligent roller was employed on the Kansas highway to enable real-time measurement of the stiffness of compacted soil, facilitating precise compaction control [122]. These instances of implementation underscore the pragmatic utilization and positive impact of IC technology in road construction projects.

The practical deployment of intelligent compaction technology over the past few decades has validated its capabilities for enhancing quality and efficiency in road construction. The following findings in this section can be drawn:

(1) Pioneering manufacturers like BOMAG, Caterpillar, Dynapac, Hamm, and Sakai have developed specialized rollers integrating precise instrumentation, automation, and connectivity solutions. Vibratory drum and padfoot variations suited different material types, achieving compaction through adjustable frequency/amplitude control. Satellite positioning, accelerometers, and thermal sensors provided real-time monitoring.
(2) Notable IC systems profiled in Section 6.2 included BOMAG’s VarioControl with rapid variable amplitude modulation and precise transmission of forces. The Ammann compaction expert ceased compaction upon reaching stiffness thresholds while documenting spatial data. Dynapac’s online management system incorporated compactness meters and analyzers with GPS integration. Meanwhile, Hammtronics and Ingersoll Rand introduced intelligent walking/excitation management and continuous density evaluation.
(3) Case studies reviewed in Section 6.3 demonstrated IC’s field-proven capabilities. For example, the Nanning–Zhanjiang Expressway project leveraged rollers integrated with the BOMAG documentation platform. Similarly, the New Cologne–Rhine/Main Line employed pedal and smooth drum machines. The TH14 Project utilized Ammann and Caterpillar rollers fitted with ACE sensors automatically adjusting energy. A Kansas highway project also featured a BOMAG intelligent system for real-time stiffness readings.
(4) While IC presents many technical advantages, its high costs and sophistication have posed barriers to widespread uptake, as discussed in Section 6.2.4. Cost–benefit analyses considering whole-lifecycle savings are therefore prudent when selecting IC solutions. Standardization efforts and larger demonstration projects can help establish code compliance procedures and specification guidelines.
(5) Some limitations encountered in IC applications include jobsite constraints hindering continuous tracking, difficult terrain disrupting positioning signals, scarce technical expertise at smaller contractor levels, and long learning curves for new technologies. Future work must address these challenges to streamline integration into mainstream practices.

In summary, IC standards and intelligent rollers have elevated compaction from labor-intensive to precisely controlled via feedback-based automation. Case applications confirm improved quality/throughput over periodic check-ups alone. Though high costs initially deter small projects, demonstrated safety/durability payoffs argue for considering IC as a preventative investment against life-cycle expenditures.

## 7. Summary and Conclusions

Over the past two decades, IC has transformed from an emerging concept into an established precision construction practice, especially within highway sectors worldwide. Driven by technological progress in areas such as sensing, communications, and control automation, IC represents a paradigm shift toward continuous, real-time management of the entire asphalt pavement compaction process. This paper provides a comprehensive review of the principles, components, developmental progress, and applications of IC technology.

The integration of specialized instrumentation like accelerometers, thermal sensors, and GNSS receivers atop modern vibratory rollers facilitates constant acquisition of quantitative metrics reflecting an asphalt mixture’s compaction response in real time. Advanced smart sensors such as SmartRock sensors, FBG sensors, and ICP acceleration sensors are also widely used in the field compaction evaluation of asphalt pavement. Through transmission over network infrastructures, perceptive data from rollers and smart sensors fuels analytical frameworks infused with ML algorithms that decode subtle indications of density, moisture, and uniformity. In turn, ML model-based controllers enforcing targeted compaction outcomes continuously modulate excitation levels automatically according to instantaneous stiffness readings inferred non-invasively.

However, in its practical implementation for asphalt pavement compaction, IC technology still encounters significant challenges that warrant focused attention from the research community. Primarily, there is a need to enhance the accuracy and reliability of data collected from pavement job sites. Environmental factors and complex terrain make it challenging to precisely assess compaction quality using current sensing and positioning techniques. Additionally, limitations in data transmission methods restrict the sharing of information between construction machinery, thereby reducing equipment flexibility and efficiency. Furthermore, the transformation of large volumes of raw data into actionable insights through processing and modeling is a complex undertaking, necessitating advanced algorithms and computing power. Lastly, expanding the application of IC to encompass diverse materials and construction scenarios also presents obstacles.

To overcome these challenges and maximize the value that IC brings to the pavement industry, concerted efforts must be devoted to the following key areas in future research:

(1) Developing Reliable Sensing and Evaluation Systems

The accuracy and reliability of sensing data serves as the cornerstone for IC’s success. Limitations of traditional sensor types highlight an urgent need for innovative sensing technology capable of withstanding harsh job site conditions. Promising solutions encompass the integration of multi-modal sensors, advanced positioning methods resistant to signal interference, and self-calibration techniques to uphold measurement accuracy over extended periods. Equally crucial is the establishment of a standardized compaction quality rating system that impartially evaluates pavement uniformity and aligns with specifications. A user-friendly rating interface would streamline quality control and optimization endeavors.

(2) Enhancing Data Collection and Transmission

The volume and diversity of data that modern sensing capacity enables far surpasses traditional practices. However, continuously collecting representative datasets remains a challenge, considering environmental variations, equipment mobility, and restrictions on sensor deployment. Emerging wireless technologies presenting opportunities, such as 5G, Bluetooth, and Wi-Fi mesh networking, could strengthen transmission robustness, coverage, and throughput to support advanced analysis. Satellite navigation backup may also boost positioning reliability. On the analysis front, edge and fog computing paradigms can distribute load and latency for real-time feedback.

(3) Advancing Data Analytics and Predictive Modeling

Vast amounts of data possess significant inherent value when subjected to thorough analysis. ML and deep learning algorithms have brought about transformative changes in numerous sectors, with their impact on the construction industry still in its early stages but potentially far-reaching. The creation of self-learning models customized for asphalt pavement compaction necessitates comprehensive field testing to guarantee predictive precision and applicability across different scenarios. Simulation methodologies could complement constrained physical testing endeavors. The establishment of standardized open formats and application programming interfaces would facilitate the swift exchange of data across projects. Progress in natural language processing and computer vision could extract valuable insights from non-numeric resources as well.

(4) Integrating with Digital Construction Technologies

The construction ecosystem consists of multiple stakeholders with diverse information needs. BIM provides a unifying framework for digital representation, coordination, simulation, and communication across disciplines and project phases. Integrating IC data seamlessly into BIM would empower authorities with a comprehensive virtual environment for planning, cost estimation, scheduling, quality control, facility management, and more. Augmented and mixed reality applications of real-time paving monitoring also show promise. Harmonizing IC with technologies like drones, robotics, IoT, and digital twins could optimize pavement workflows end-to-end.

(5) Validating Models and Systems through Field Projects

Field testing and validation remain essential to bridge the gap between lab research and implementation challenges in diverse real-world settings. Larger-scale demonstration projects provide opportunities for stakeholders to experience IC’s usefulness firsthand, establish code and specification standards, and provide feedback for continual enhancements. Developing an open testbed network would facilitate benchmark evaluations and cross-fertilization of ideas. Public–private partnerships can also sponsor pilot implementations and deployment pathways. Ultimately, strengthening industry–academia collaborations will be instrumental in addressing complex practical challenges through joint knowledge dissemination and problem solving.

In conclusion, IC represents a transformative paradigm that upgrades asphalt pavement construction to meet the precision demands of sustainable infrastructure development. While significant milestones have been achieved, numerous technical and performance barriers still necessitate multidisciplinary cooperation. With concerted efforts channeled towards reliable sensing, ubiquitous connectivity, predictive analytics, digital integration, as well as validation, verification, and demonstration, IC’s full potential to revolutionize quality control practices and reshape our road networks can be realized.

## Figures and Tables

**Table 1:** U.S. Intelligent compaction specifications.

Organization	Standard Name
FHWA	Intelligent Compaction Technology for Asphalt Application. 2014
AASHTO	Standard Practice for Intelligent Compaction Technology for Embankment and Asphalt Applications
AASHTO	Intelligent Compaction Technology for HMA Applications. Generic-IC Specification for HMA. June, 2011
AASHTO	Intelligent Compaction Technology for Embankment and Asphalt Pavement Applications

**Table 2:** Comparison results of different compaction techniques.

Compare Options	Traditional Compaction Technology	IC Technology
Quality control	Relying on manual inspection and laboratory testing, relying on a few sample points, cannot fully represent the compaction situation of the entire construction area.	Equipped with sensors and real-time feedback, it can monitor the compaction status of the entire construction surface, provide continuous quality control and real-time optimization suggestions.
Rolling thickness	Unable to record the starting pile number, manual control, inaccurate recording of layered rolling thickness.	Accurately records the starting pile number and roll thickness of each layer, and precisely controls the compaction work. The system will consider soil type, humidity, and other variables to obtain the best results.
Data recording	Manual operation is prone to errors, and the recorded data may be incomplete, which cannot meet the recording requirements of the entire construction process data.	Capable of real-time recording and archiving of data such as rolling frequency and quality, optimizing construction record management.
Data display	The data display lags behind and cannot display the current compaction degree in real time.	Records and displays data in real time throughout the entire process, and promptly identifies and marks unqualified construction areas.
Construction cost	Low cost and stable price.	The cost of a single project may be 3% to 5% higher, but in the long run, improving efficiency may offset the high costs.

**Table 3:** Comparison of three sensors.

Sensor	Advantage	Disadvantages
SmartRock sensors	Real-time monitoring. No need to dismantle or destroy the structure. Accurate data. Cost saving.	Dependent technology: users may need specific skills and knowledge to operate and understand data. Limited monitoring scope and depth. Power supply required.
FBG sensors	High precision. Strong anti-interference ability. Multi parameter measurement. Miniaturization and lightweight. Long-term stability.	High cost. Require special equipment: additional incentives and measuring equipment are required. Temperature bias problem.
ICP acceleration sensor	High sensitivity. Wide frequency response range. Simplified measurement system. High measurement accuracy.	Power dependent. More complex to deploy and maintain. High cost.

**Table 4:** Advantages and disadvantages of different compaction quality evaluation indicators.

Compaction Quality Evaluation Index	Advantage	Disadvantages
CMV	Can intuitively reflect the soil compaction degree. Quantitative comparison. Simple operation, do not need sophisticated instruments and equipment.	Lack of comprehensiveness: it can only reflect the hardness of the soil to a certain extent. Affected by external factors. Not suitable for different compression scenarios.
CCV	Rapid and economic. Quantitative indicators, evaluation is more objective and systematic. Can be used for comparative analysis.	Depends on the maximum dry density. Cannot reflect the engineering properties of materials.
MDP	High reliability. Real-time measurement during actual compaction. Wide application.	High equipment cost. Need a specific device and sensors. Cannot fully reflect the compaction situation. Affected by terrain.
AICV	Comprehensive: can comprehensively evaluate the compaction quality. Real-time monitoring with timely feedback and adjustment. Has certain adaptability. Increased safety.	High cost. Technical complexity. Special or complex terrain conditions, need human intervention. Generate a certain amount of energy consumption and emissions. Accuracy depends on the precision and reliability of the sensors used.

**Table 5:** Manufacturers and technical parameters of intelligent road roller for soil compaction.

Manufacturer	Case	BOMAG	Caterpillar	Dynapac	Sakai
Technology	Compaction experts and dynamic analyzers	Automatic amplitude modulation compaction	Numerical analyzer	Digital communications analyzer, global positioning system	Exact compact
Automatic feedback system	Yes	Yes	Yes	Yes	Yes
Measuring system	Yes	Yes	Yes	Yes	Yes
Measurement (units)	Stiffness (MN/m)	Vibration modulus (MN/m^2^)	Compactness (/)	Compactness (/)	Compact control (/)
Global positioning capability	Yes	Yes	Yes	Yes	Yes
Storage system	Compaction experts and dynamic analyzers	Compaction management system and hard disk	Laser control system	Digital communication analyzer	Numerical analyzer

**Table 6:** Manufacturers and technical parameters of intelligent roller for asphalt concrete compaction.

Manufacturer	Case	BOMAG	Caterpillar	Dynapac	Sakai	Volvo
Technology	Numerical analyzer	Asphalt manager	Laser control system	Digital communication analyzer	Exact compact	TBA
Automatic feedback system	Numerical analyzer	Yes	/	Yes.	/	TBA
Measuring system	Numerical analyzer	Yes	Yes	Yes	Yes	Yes
Measurement (units)	/	Vibration modulus (MN/m^2^)	Temperature (°C)	Compactness (/)	Compactness (/)	TBA
Global positioning capability	Numerical analyzer	Yes	Yes	Yes	Yes	Yes
Storage system	Numerical analyzer	Compaction management system and hard disk	Laser control system	Digital communication analyzer	Aithon MT-R system	TBA

**Table 7:** Advantages and disadvantages of different compaction systems.

Intelligent Compaction System	Advantages	Disadvantages
BOMAG VARIOCONTROL	Automatic amplitude variation. High working efficiency. Precision compaction.	Higher costs. Complex operation. Professional training required, there is a risk of technical failure.
AMMANN compaction expert	Efficient, real-time monitoring and analysis of the compaction process. Ability to precisely control compaction parameters. Automation, reducing the need for manual intervention and the possibility of human error. Real-time recording and storage of data from the compaction process.	High investment and operating costs Technical malfunctions may cause interruptions.
DYNAPAC single drum vibratory roller online compaction management system	Real-time monitoring and analysis of compaction data. Real-time data collection. Advance warning of mechanical faults.	High cost, higher investment. Technical complexity. High equipment dependency.
HAMMTRONIC	Highly automated. Ensures uniform compaction on different sections. Real-time data monitoring. Reduced labor dependency.	High investment costs. High technical requirements. Fault maintenance issues may occur frequently.
Ingersoll Rand compactness test system	Provides reliable performance and excellent compaction results. Equipped with advanced technology and features to improve construction efficiency.	High investment costs. High technical threshold. There is a risk of maintenance and malfunction.

## Data Availability

The raw data supporting the conclusions of this article will be made available by the authors upon request.

## References

[1] Gao Y., Huang X., Yu W. (2014). The compaction characteristics of hot mixed asphalt mixtures. J. Wuhan Univ. Technol. Mater. Sci. Ed..

[2] Li J., Qin Y., Zhang X., Shan B., Liu C. (2024). Emission Characteristics, Environmental Impacts, and Health Risks of Volatile Organic Compounds from Asphalt Materials: A State-of-the-Art Review. Energy Fuels.

[3] Zhou S., Yan J., Shi B., Li S., Ai C., Yan C. (2023). Green synthesis of a broad-spectrum UV-blocking bitumen modifier: Investigation of anti-aging performance and mechanism in bitumen. J. Clean. Prod..

[4] Zhou S., Li S., Yan C.J.C., Materials B. (2023). Influence of fumed silica nanoparticles on the rheological and anti-aging properties of bitumen. Constr. Build. Mater..

[5] Winter M.G. (2020). Continuous compaction control in the UK: History, current state and future prognosis. Proc. Inst. Civ. Eng. Geotech. Eng..

[6] Liu P., Hu J., Falla G.C., Wang D., Leischner S., Oeser M.J.C., Materials B. (2019). Primary investigation on the relationship between microstructural characteristics and the mechanical performance of asphalt mixtures with different compaction degrees. Constr. Build. Mater..

[7] Zhang Q., An Z., Huangfu Z., Li Q. (2022). A review on roller compaction quality control and assurance methods for earthwork in five application scenarios. Materials.

[8] Jian-zhong P. (2018). Progress of highway engineering and generation upgrading of highway transportation system. China J. Highw. Transp..

[9] Hu W., Shu X., Huang B., Woods M. (2017). Field investigation of intelligent compaction for hot mix asphalt resurfacing. Front. Struct. Civ. Eng..

[10] Liu D., Wang Y., Chen J., Zhang Y. (2020). Intelligent compaction practice and development: A bibliometric analysis. Eng. Constr. Arch. Manag..

[11] Cai J., Gao Q., Chun H., Cai H., Nantung T. (2019). Spatial autocorrelation in soil compaction and its impact on earthwork acceptance testing. Transp. Res. Rec..

[12] Xu Q., Chang G.K. (2016). Adaptive quality control and acceptance of pavement material density for intelligent road construction. Autom. Constr..

[13] Xu Q., Chang G.K. (2013). Evaluation of intelligent compaction for asphalt materials. Autom. Constr..

[14] Barman M., Nazari M., Imran S.A., Commuri S., Zaman M., Beainy F., Singh D. (2016). Quality control of subgrade soil using intelligent compaction. Innov. Infrastruct. Solut..

[15] Sivagnanasuntharam S., Sounthararajah A., Ghorbani J., Bodin D., Kodikara J. (2023). A state-of-the-art review of compaction control test methods and intelligent compaction technology for asphalt pavements. Road Mater. Pavement Des..

[16] Horan R.D., Chang G.K., Xu Q., Gallivan V.L. (2012). Improving quality control of hot-mix asphalt paving with intelligent compaction technology. Transp. Res. Rec..

[17] Bacci di Capaci R., Scali C. (2020). A cloud-based monitoring system for performance assessment of industrial plants. Ind. Eng. Chem. Res..

[18] Gallivan V.L., Chang G.K., Horan D.R. (2011). Intelligent compaction for improving roadway construction. Emerging Technologies for Material, Design, Rehabilitation, and Inspection of Roadway Pavements.

[19] Tanoli W.A., Seo J.W., Sharafat A., Lee S.S. (2018). 3D design modeling application in machine guidance system for earthwork operations. KSCE J. Civ. Eng..

[20] Sharafat A., Khan M.S., Latif K., Seo J. (2021). BIM-based tunnel information modeling framework for visualization, management, and simulation of drill-and-blast tunneling projects. J. Comput. Civ. Eng..

[21] Cui X., Li X., Du Y., Bao Z., Zhang X., Hao J., Hu Y.J.C., Materials B. (2024). Macro-micro numerical analysis of granular materials considering principal stress rotation based on DEM simulation of dynamic hollow cylinder test. Constr. Build. Mater..

[22] Xu G., Wang D. (2022). Introduction to Intelligent Construction Technology of Transportation Infrastructure.

[23] Chen S., Liu X., Luo H., Yu J., Chen F., Zhang Y., Ma T., Huang X. (2022). A state-of-the-art review of asphalt pavement surface texture and its measurement techniques. J. Road Eng..

[24] Hu W., Shu X., Huang B., Woods M.E. (2018). An examination of compaction meter value for asphalt pavement compaction evaluation. Int. J. Pavement Eng..

[25] Hu W., Jia X., Zhu X., Gong H., Xue G., Huang B. (2019). Investigating key factors of intelligent compaction for asphalt paving: A comparative case study. Constr. Build. Mater..

[26] Ling J., Lin S., Qian J., Zhang J., Han B., Liu M. (2018). Continuous compaction control technology for granite residual subgrade compaction. J. Mater. Civ. Eng..

[27] Jia T., He T., Qian Z., Lv J., Cao K. (2019). An improved low-cost continuous compaction detection method for the construction of asphalt pavement. Adv. Civ. Eng..

[28] Kumar S.A., Aldouri R., Nazarian S., Si J. (2016). Accelerated assessment of quality of compacted geomaterials with intelligent compaction technology. Constr. Build. Mater..

[29] Savan C.M., Ng K.W., Ksaibati K. (2016). Benefit-cost analysis and application of intelligent compaction for transportation. Transp. Geotech..

[30] Chang G.K., Mohanraj K., Stone W.A., Oesch D.J., Gallivan V. (2018). Leveraging intelligent compaction and thermal profiling technologies to improve asphalt pavement construction quality: A case study. Transp. Res. Rec..

[31] Zhang Q., Liu T., Zhang Z., Huangfu Z., Li Q., An Z. (2019). Unmanned rolling compaction system for rockfill materials. Autom. Constr..

[32] Wang L., Wang H., Zhao Q., Yang H., Zhao H., Huang B. (2019). Development and prospect of intelligent pavement. China J. Highw. Transp..

[33] Zhu X., Bai S., Xue G., Yang J., Cai Y., Hu W., Jia X., Huang B. (2018). Assessment of compaction quality of multi-layer pavement structure based on intelligent compaction technology. Constr. Build. Mater..

[34] Wu J., Luo Z., Xu G. (2017). Progress of research on intelligent compaction technology. Road Mach. Constr. Mech..

[35] Xu G., Luo Z., Tian B. (2015). Summary of development of continuous compaction control technology. Road Mach. Constr. Mech..

[36] Xu Q., Chang G.K., Gallivan V.L. (2012). Development of a systematic method for intelligent compaction data analysis and management. Constr. Build. Mater..

[37] Nazarian S., Fathi A., Tirado C., Kreinovich V., Rocha S., Mazari M. (2020). Evaluating Mechanical Properties of Earth Material during Intelligent Compaction.

[38] Chang G., Gallivan V.L. (2011). Accelerated Implementation of Intelligent Compaction Technology for Embankment Subgrade Soils, Aggregate Base, and Asphalt Pavement Materials.

[39] Meehan C.L., Cacciola D.V., Tehrani F.S., Baker W.J. (2017). Assessing soil compaction using continuous compaction control and location-specific in situ tests. Autom. Constr..

[40] Ma J., Sun S., Rui H., Wang L., Ma Y., Zhang W., Zhang W., Liu H., Chen H., Liu J. (2018). Review on China’s road construction machinery research progress: 2018. China J. Highw. Transp..

[41] Yu H., Ma T., Wang D., Wang Z., Lv S., Zhu X. (2020). Review on China’s pavement engineering research 2020. China J. Highw. Transp.

[42] Zhang Q., Liu T., Zhang Z., Huangfu Z., Li Q., An Z. (2019). Compaction quality assessment of rockfill materials using roller-integrated acoustic wave detection technique. Autom. Constr..

[43] Chen B., Yu X., Dong F., Zheng C., Ding G., Wu W. (2021). Compaction quality evaluation of asphalt pavement based on intelligent compaction technology. Constr. Eng. Manag..

[44] Nedoma J., Stolarik M., Kepak S., Pinka M., Martinek R., Frnda J., Fridrich M.J.S. (2019). Alternative Approaches to Measurement of Ground Vibrations Due to the Vibratory Roller: A Pilot Study. Sensors.

[45] Ma Y., Zhang Y., Zhao W., Ding X., Wang Z., Ma T. (2022). Assessment of intelligent compaction quality evaluation index and uniformity. J. Transp. Eng. Part B Pavements.

[46] Wang X., Shen S., Huang H., Zhang Z. (2019). Towards smart compaction: Particle movement characteristics from laboratory to the field. Constr. Build. Mater..

[47] Ma Y., Chen F., Ma T., Huang X., Zhang Y. (2021). Intelligent compaction: An improved quality monitoring and control of asphalt pavement construction technology. IEEE Trans. Intell. Transp. Syst..

[48] Xu G., Chang G.K., Wang D., Correia A.G., Nazarian S. (2022). The pioneer of intelligent construction—An overview of the development of intelligent compaction. J. Road Eng..

[49] Xu G., Gao H., Luo Z., Huang J., Wang D.S. (2017). Application of continuous and intelligent compaction control technology in high-speed railway construction. Road Mach. Constr. Mech..

[50] Ranasinghe R., Sounthararajah A., Kodikara J. (2023). An Intelligent Compaction Analyzer: A versatile platform for real-time recording, monitoring, and analyzing of road material compaction. Sensors.

[51] Han T., Ma T., Fang Z., Zhang Y., Han C. (2022). A BIM-IoT and intelligent compaction integrated framework for advanced road compaction quality monitoring and management. Comput. Electr. Eng..

[52] Sivagnanasuntharam S., Sounthararajah A., Kodikara J. (2023). A new approach to maximising the benefits of current intelligent compaction technology for asphalt materials. Constr. Build. Mater..

[53] Yao Y., Song E. (2023). Intelligent compaction methods and quality control. Smart Constr. Sustain. Cities.

[54] Chen L., Ghorbani J., Tophel A., Kodikara J. An unsaturated soil mechanics approach for performance-based intelligent compaction. Proceedings of the E3S Web of Conferences.

[55] Chen L., Ghorbani J., Zhang C., Dutta T., Kodikara J. A constitutive modelling approach towards performance-based intelligent compaction. Proceedings of the Supplement to the Proceedings of the International Society for Intelligent Construction 2022 Conference (ISIC 2022).

[56] Wang N., Ma T., Chen F., Ma Y. (2022). Compaction quality assessment of cement stabilized gravel using intelligent compaction technology—A case study. Constr. Build. Mater..

[57] Chen Y., Yu Q., Li W., Xiao Y., Yang T., Li Z., Zhi X., Deng P. (2022). Experimental Study on Vibratory Compaction Behavior of Tunneling Rock Wastes Used as Unbound Permeable Aggregate Base Materials. Materials.

[58] Zhang T., Zheng Y., Wang C., Mu Z., Liu Y., Lin J. (2018). A review of photonic crystal fiber sensor applications for different physical quantities. Appl. Spectrosc. Rev..

[59] Kirianaki N.V., Yurish S.Y., Shpak N.O., Deynega V.P. (2002). Data Acquisition and Signal Processing for Smart Sensors.

[60] Wang X., Shen S., Huang H., Almeida L.C. (2018). Characterization of particle movement in Superpave gyratory compactor at meso-scale using SmartRock sensors. Constr. Build. Mater..

[61] De Maeijer P.K., Bergh W.V.D., Vuye C. (2018). Fiber Bragg Grating Sensors in Three Asphalt Pavement Layers. Infrastructures.

[62] Hemmat A., Adamchuk V. (2008). Sensor systems for measuring soil compaction: Review and analysis. Comput. Electron. Agric..

[63] Tang K., Yuan H., Lv J., Chen F. (2020). Research on the Method for Analyzing the Degree of Impact Acceleration and Compaction of the Impact Roller. IEEE Access.

[64] Liu S., Huang H., Qiu T., Gao L. (2017). Comparison of Laboratory Testing Using SmartRock and Discrete Element Modeling of Ballast Particle Movement. J. Mater. Civ. Eng..

[65] Liu S., Qiu T., Qian Y., Huang H., Tutumluer E., Shen S. (2019). Simulations of large-scale triaxial shear tests on ballast aggregates using sensing mechanism and real-time (SMART) computing. Comput. Geotech..

[66] Wang X., Huang H., Tutumluer E., Tingle J.S., Shen S. (2022). Monitoring Particle Movement under Compaction using SmartRock Sensor: A Case Study of Granular Base Layer Compaction. Transp. Geotech..

[67] Yu S., Shen S. (2022). Compaction Prediction for Asphalt Mixtures Using Wireless Sensor and Machine Learning Algorithms. IEEE Trans. Intell. Transp. Syst..

[68] Liu S., Huang H., Qiu T., Kwon J. (2016). Effect of geogrid on railroad ballast particle movement. Transp. Geotech..

[69] Yu S., Shen S., Lu M. (2023). Data sensing and compaction condition modeling for asphalt pavements. Autom. Constr..

[70] Wang X., Shen S., Huang H., Yu S. (2023). Understanding the role of particle rotation in asphalt mixture compaction by tracking coarse aggregate movement. Constr. Build. Mater..

[71] Liao M., Liang S., Luo R., Chen Y. (2023). The moving load identification method on asphalt roads based on the BP neural network and FBG sensor monitoring. Constr. Build. Mater..

[72] Wang H., Xiang P., Jiang L. (2018). Optical Fiber Sensor Based In-Field Structural Performance Monitoring of Multilayered Asphalt Pavement. J. Light. Technol..

[73] Bado M.F., Casas J.R. (2021). A Review of Recent Distributed Optical Fiber Sensors Applications for Civil Engineering Structural Health Monitoring. Sensors.

[74] Li C.Z., Guo Z., Su D., Xiao B., Tam V.W.Y. (2022). The Application of Advanced Information Technologies in Civil Infrastructure Construction and Maintenance. Sustainability.

[75] Pendão C., Silva I. (2022). Optical Fiber Sensors and Sensing Networks: Overview of the Main Principles and Applications. Sensors.

[76] Sahota J.K., Gupta N., Dhawan D. (2020). Fiber Bragg grating sensors for monitoring of physical parameters: A comprehensive review. Opt. Eng..

[77] Her S.-C., Lin W.-N. (2020). Simultaneous Measurement of Temperature and Mechanical Strain Using a Fiber Bragg Grating Sensor. Sensors.

[78] Bhaskar C.V.N., Pal S., Pattnaik P.K. (2021). Recent advancements in fiber Bragg gratings based temperature and strain measurement. Results Opt..

[79] Wang H., Xiang P., Jiang L. (2019). Strain transfer theory of industrialized optical fiber-based sensors in civil engineering: A review on measurement accuracy, design and calibration. Sens. Actuators A Phys..

[80] Cao Y.W., Jiao J.H., Ma L.Y., Gui S.X. (2011). Research on the ICP Acceleration Sensors Detecting Roadbed Compaction Degree Test. Adv. Mater. Res..

[81] Yiqiu T., Haipeng W., Shaojun M., Huining X. (2014). Quality control of asphalt pavement compaction using fibre Bragg grating sensing technology. Constr. Build. Mater..

[82] Wang N., Chen F., Ma T., Luan Y., Zhu J. (2022). Compaction performance of cold recycled asphalt mixture using SmartRock sensor. Autom. Constr..

[83] Liu D., Lin M., Li S. (2016). Real-Time Quality Monitoring and Control of Highway Compaction. Autom. Constr..

[84] Minchin Jr R.E., Thomas H.R. (2003). Validation of vibration-based onboard asphalt density measuring system. J. Constr. Eng. Manag..

[85] Dan H.-C., Yang D., Liu X., Peng A.-P., Zhang Z. (2020). Experimental investigation on dynamic response of asphalt pavement using SmartRock sensor under vibrating compaction loading. Constr. Build. Mater..

[86] White D.J., Thompson M.J. (2008). Relationships between In Situ and Roller-Integrated Compaction Measurements for Granular Soils. J. Geotech. Geoenviron. Eng..

[87] Cai H., Kuczek T., Dunston P.S., Li S. (2017). Correlating intelligent compaction data to in situ soil compaction quality measure-ments. J. Constr. Eng. Manag..

[88] Yao Y., Zhang X., Wang Z., Cao S., Ma X. (2023). Compaction quality evaluation method based on dual-index in intelligent com-paction of filling foundation. Transp. Geotech..

[89] Liu D., Li Z., Lian Z. (2014). Compaction quality assessment of earth-rock dam materials using roller-integrated compaction moni-toring technology. Autom. Constr..

[90] Sandström A.J., Pettersson C.B. Intelligent systems for QA/QC in soil compaction. Proceedings of the 83rd Annual Transportation Research Board Meeting.

[91] Chen J., Huang B., Shu X., Hu C. (2015). DEM Simulation of Laboratory Compaction of Asphalt Mixtures Using an Open Source Code. J. Mater. Civ. Eng..

[92] Taffese W.Z., Abegaz K.A. (2022). Prediction of Compaction and Strength Properties of Amended Soil Using Machine Learning. Buildings.

[93] Xu Z., Khabbaz H., Fatahi B., Wu D. (2022). Real-time determination of sandy soil stiffness during vibratory compaction incorporating machine learning method for intelligent compaction. J. Rock Mech. Geotech. Eng..

[94] Isik F., Ozden G. (2013). Estimating compaction parameters of fine- and coarse-grained soils by means of artificial neural networks. Environ. Earth Sci..

[95] Shen S., Wang L., Zhang C., Ildefonzo D. (2022). Use of SmartRock Sensors to Monitor Pavement Condition for Supporting Maintenance Decision Making.

[96] Shi X., Kang Q., An J., Zhou M. (2021). Novel L1 Regularized Extreme Learning Machine for Soft-Sensing of an Industrial Process. IEEE Trans. Ind. Inform..

[97] Shao Z., Zhao R., Yuan S., Ding M., Wang Y. (2022). Tracing the evolution of AI in the past decade and forecasting the emerging trends. Expert Syst. Appl..

[98] Acharya U., Daigh A.L.M., Oduor P.G. (2021). Machine Learning for Predicting Field Soil Moisture Using Soil, Crop, and Nearby Weather Station Data in the Red River Valley of the North. Soil Syst..

[99] Rajan M.P. (2022). An Efficient Ridge Regression Algorithm with Parameter Estimation for Data Analysis in Machine Learning. SN Comput. Sci..

[100] Assegie T.A., Salau A.O., Badrudeen T.U. (2022). Estimation of concrete compression using regression models. Bull. Electr. Eng. Informatics.

[101] Charbuty B., Abdulazeez A. (2021). Classification based on decision tree algorithm for machine learning. J. Appl. Sci. Technol. Trends.

[102] Mao C., Lu L., Hu B. (2020). Local probabilistic model for Bayesian classification: A generalized local classification model. Appl. Soft Comput..

[103] Sen P.C., Hajra M., Ghosh M. (2020). Supervised classification algorithms in machine learning: A survey and review. Emerging Technology in Modelling and Graphics: Proceedings of IEM Graph 2018.

[104] Mohammed A., Kora R. (2023). A comprehensive review on ensemble deep learning: Opportunities and challenges. J. King Saud Univ. Comput. Inf. Sci..

[105] Diao W., Liu G., Zhang H., Hu K., Jin X. (2021). Influences of Soil Bulk Density and Texture on Estimation of Surface Soil Moisture Using Spectral Feature Parameters and an Artificial Neural Network Algorithm. Agriculture.

[106] Gong J., Yu Y., Krishnamoorthy R., Roda A. (2015). Real-time tracking of concrete vibration effort for intelligent concrete consoli-dation. Autom. Constr..

[107] Lee S.G., Skibniewski M.J. Monitoring of concrete placement and vibration for real-time quality control. Proceedings of the Creative Construction Conference 2019.

[108] Cheng C., Shen Z. (2021). Semi real-time detection of subsurface consolidation defects during concrete curing stage. Constr. Build. Mater..

[109] Fathi A., Tirado C., Rocha S., Mazari M., Nazarian S. (2021). A Machine-Learning Approach for Extracting Modulus of Compacted Unbound Aggregate Base and Subgrade Materials Using Intelligent Compaction Technology. Infrastructures.

[110] Pereira T.d.S., Robaina A.D., Peiter M.X., Torres R.R., Bruning J. (2018). The use of artificial intelligence for estimating soil resistance to penetration. Eng. Agrícola.

[111] Wang X., Dong X., Zhang Z., Zhang J., Ma G., Yang X. (2022). Compaction quality evaluation of subgrade based on soil characteristics assessment using machine learning. Transp. Geotech..

[112] Chen C., Hu Y., Jia F., Wang X. (2022). Intelligent compaction quality evaluation based on multi-domain analysis and artificial neural network. Constr. Build. Mater..

[113] Wang J., Zhong D., Adeli H., Wang D., Liu M. (2018). Smart bacteria-foraging algorithm-based customized kernel support vector regression and enhanced probabilistic neural network for compaction quality assessment and control of earth-rock dam. Expert Syst..

[114] Zhan Y., Zhang Y., Nie Z., Luo Z., Qiu S., Wang J., Zhang A.A., Ai C., Tang X., Tan C. (2023). Intelligent paving and compaction technologies for asphalt pavement. Autom. Constr..

[115] Pilataxi Araujo T. (2021). Paving and Compaction Support Systems. The Status of Implementation Worldwide. Bachelor’s Thesis.

[116] Wang S., Sui X., Leng Z., Jiang J., Lu G. (2022). Asphalt pavement density measurement using non-destructive testing methods: Current practices, challenges, and future vision. Constr. Build. Mater..

[117] Makarov D., Miller S., Vahdatikhaki F., Dorée A. (2019). Comprehensive real-time pavement operation support system using machine-to-machine communication. Int. J. Pavement Res. Technol..

[118] Erdmann P., Adam D. Numerical simulation of dynamic soil compaction with vibratory compaction equipment. Proceedings of the Geotechnics of Roads and Railways: Proceedings XV Danube–European Conference on Geotechnical Engineering.

[119] Singh R. (2018). Model-based control system design and evaluation for continuous tablet manufacturing processes (via direct com-paction, via roller compaction, via wet granulation). Computer Aided Chemical Engineering.

[120] Briaud J.-L., Seo J. (2003). Intelligent compaction: Overview and research needs. Tex. AM Univ..

[121] Camargo F., Larsen B., Chadbourn B., Roberson R., Siekmeier J. Intelligent compaction: A Minnesota case history. Proceedings of the 54th Annual University of Minnesota Geotechnical Conference.

[122] Rahman F., Hossain M., Romanoschi S., Brennan J. (2012). Kansas experience with stiffness-based quality control/quality assurance specifications for compaction of highway embankments. GeoCongress 2012: State of the Art and Practice in Geotechnical Engineering.

